# iPSC-derived cells lack immune tolerance to autologous NK-cells due to imbalance in ligands for activating and inhibitory NK-cell receptors

**DOI:** 10.1186/s13287-023-03308-5

**Published:** 2023-04-11

**Authors:** Margarita E. Bogomiakova, Elizaveta K. Sekretova, Ksenia S. Anufrieva, Polina O. Khabarova, Anastasia N. Kazakova, Pavel A. Bobrovsky, Tatiana V. Grigoryeva, Artem V. Eremeev, Olga S. Lebedeva, Alexandra N. Bogomazova, Maria A. Lagarkova

**Affiliations:** 1 Lopukhin Federal Research and Clinical Center of Physical-Chemical Medicine of Federal Medical Biological Agency, 1a Malaya Pirogovskaya, Moscow, Russia 119435; 2 Faculty of Biology, Lomonosov Moscow State University, 1-12 Leninskie Gory, Moscow, Russia 119991; 3 Center for Precision Genome Editing and Genetic Technologies for Biomedicine, Lopukhin Federal Research and Clinical Center of Physical-Chemical Medicine of Federal Medical Biological Agency, 1a Malaya Pirogovskaya, Moscow, Russia 119435; 4 Kazan Federal University, 18 Kremlyovskaya Street, Kazan, Russia 420008

**Keywords:** Autologous iPSCs, NK-cells, Differentiation, HLA-I, Beta-2-microglobulin, Immunogenicity, Immune tolerance

## Abstract

**Background:**

Dozens of transplants generated from pluripotent stem cells are currently in clinical trials. The creation of patient-specific iPSCs makes personalized therapy possible due to their main advantage of immunotolerance. However, some reports have claimed recently that aberrant gene expression followed by proteome alterations and neoantigen formation can result in iPSCs recognition by autologous T-cells. Meanwhile, the possibility of NK-cell activation has not been previously considered. This study focused on the comparison of autologous and allogeneic immune response to iPSC-derived cells and isogeneic parental somatic cells used for reprogramming.

**Methods:**

We established an isogeneic cell model consisting of parental dermal fibroblasts, fibroblast-like iPSC-derivatives (iPS-fibro) and iPS-fibro lacking beta-2-microglobulin (B2M). Using the cells obtained from two patients, we analyzed the activation of autologous and allogeneic T-lymphocytes and NK-cells co-cultured with target cells.

**Results:**

Here we report that cells differentiated from iPSCs can be recognized by NK-cells rather than by autologous T-cells. We observed that iPS-fibro elicited a high level of NK-cell degranulation and cytotoxicity, while isogeneic parental skin fibroblasts used to obtain iPSCs barely triggered an NK-cell response. iPSC-derivatives with *B2M* knockout did not cause an additional increase in NK-cell activation, although they were devoid of HLA-I, the major inhibitory molecules for NK-cells. Transcriptome analysis revealed a significant imbalance of ligands for activating and inhibitory NK-cell receptors in iPS-fibro. Compared to parental fibroblasts, iPSC-derivatives had a reduced expression of HLA-I simultaneously with an increased gene expression of major activating ligands, such as *MICA*, *NECTIN2*, and *PVR*. The lack of inhibitory signals might be due to insufficient maturity of cells differentiated from iPSCs. In addition, we showed that pretreatment of iPS-fibro with proinflammatory cytokine IFNγ restored the ligand imbalance, thereby reducing the degranulation and cytotoxicity of NK-cells.

**Conclusion:**

In summary, we showed that iPSC-derived cells can be sensitive to the cytotoxic potential of autologous NK-cells regardless of HLA-I status. Thus, the balance of ligands for NK-cell receptors should be considered prior to iPSC-based cell therapies.

*Trial registration* Not applicable.

**Supplementary Information:**

The online version contains supplementary material available at 10.1186/s13287-023-03308-5.

## Background

The recent progress in the field of human pluripotent stem cells (hPSCs) [1, 2] and advances in organ bioengineering [3, 4] demonstrate significant potential for the development of regenerative medicine. The induced pluripotent stem cells (iPSCs) and embryonic stem cells (ESCs) both have the potential to differentiate into all cell types in the body, and this gives great hope for solving the problem of the shortage of donor organs [5]. According to clinicaltrials.gov, more than 50 clinical studies on hPSCs are being conducted. While most of them are performed with ESC-derived cell products, the number of iPSC-based studies has significantly increased in the last few years [6].

One of major barriers to integrating hPSCs into the clinic is its high cost. According to a recent estimate, the derivation of a clinical grade iPSCs line costs approximately US $800,000 [7]. The long time required to obtain a new iPSCs line, as well as to differentiate it into the desired cell type, is also worthy of emphasis [8]. Additionally, there are currently no clearly developed standardization parameters that would be applied to clinical-grade hPSCs [9] and their differentiated derivatives [10]. Thus, cell products derived from only five well-characterized ESCs lines were used in almost half of hPSC-based studies [9]. In this regard, standardized hPSC-derivatives are currently considered as the preferred source of cells for replacement therapy.

Despite the obvious economic advantages regarding the production and "scaling-up" of allogeneic hPSC-derivatives, the issue of immune rejection remains unresolved. As such, after transplantation of allogeneic hPSC-derivatives, patients must undergo lifelong immunosuppressive treatment, along with its associated side effects [10]. An alternative way to reduce the rejection of an allotransplant is creating an immunoengineered hPSCs devoid of major immune antigens that trigger immune responses [11]. Such hypoimmunogenic cells will be “universal,” meaning that they would be theoretically suitable for any recipient [12, 13]. The diversity of the HLA phenotypes is the main reason for histoincompatibility. As such, HLA-editing is the most common approach to creating hypoimmunogenic hPSCs lines, with the most common modification—the knockout of a light chain of HLA-I dimer encoded by the *beta-2-microglobulin* (*B2M*) gene [14–21]. In some studies, individual genes of the HLA-I locus were edited [22–25]. As a result, HLA-deficient cells evoked a blunted allogeneic T-cell response in recipients [13, 14, 26]. In addition, several reports described the double knockout of the *B2M* gene and the *CIITA* gene coding class II major histocompatibility complex transactivator [27–31]. The latest is essential for HLA-II expression. Since HLA class II molecules are expressed by a limited type of cells, mostly antigen-presenting cells, the *CIITA* deficiency reduces the responses of allogeneic CD4^+^ T-cells and could be beneficial for the transplantation of vascularized organs [32].

On the other hand, the absence of HLA-I molecules can trigger increased NK-cell lysis in recipients [12–14, 26]. The conventional “missing-self” hypothesis proposes that NK-cells recognize and eliminate all cells lacking HLA class I molecules [33]. The modern view of this theory is more complex and involves the interaction between a diversity of activating and inhibitory receptors on NK-cells [34]. The integration of both activating and inhibitory signals coming from the corresponding receptors regulates NK-cell activity. A preponderance in one direction or another leads to a change of the functional behavior of NK-cells. Therefore, it is possible to manipulate NK-cell responses by shifting the balance toward their inhibition. Among the strategies for escaping the immune attack mediated by NK-cells, the ectopic expression of the immunomodulation molecules such as HLA-E [31, 35], HLA-G [18, 23], and CD47 molecules [23, 28] was examined. Additional suppression of NK-cell activity can be achieved by the inactivation of NK-cell activating receptors [31].

Immune evasion reduces the risk of rejection of allogeneic hPSC-derivatives but weakens the host's defense against the possible oncogenic transformation of cells in the graft [36]. Moreover, the biosafety and effectiveness of "universal" cell lines have yet to be proven. Finally, the role of HLA molecules is essential in the efficient immune response to virus-infected cells [37].

Autologous iPSCs and their derivatives were initially perceived as immunologically tolerant [38]. Surprisingly, some reports pointed to the immune response toward syngeneic and autologous iPSC-derivatives. Zhao et al*.* revealed T-cell infiltration zones in teratomas formed in mice by syngeneic iPSCs but not ESCs [39]. In the humanized mouse model, teratomas from autologous iPSCs had signs of lymphocytic infiltration and necrosis [40]. It was assumed that neoepitopes that can arise during reprogramming triggered the immune response to autologous iPSC-derived cells [41]. For instance, de novo mutations in mitochondrial DNA in iPSCs produced immunogenic neoepitopes in mice [42]. Thus, there are reports that autologous iPSC-derived cells are recognized by the immune system, although such reports are not numerous.

The goal of our study was to address the question whether autological T-cells and NK-cells recognize the differentiated derivatives of iPSCs as “self.” We established an isogenic cell model: dermal fibroblasts, iPSC-derived fibroblast-like cells (iPS-fibro), and iPS-fibro with the knockout of the *B2M* gene (ΔiPS-fibro). Using this model, we compared in vitro activation of allogeneic and autologous immune cells. It is worth noting that our study was made possible because we had the rare opportunity to obtain iPSCs from healthy donors who also agreed to periodically donate small amounts of peripheral blood to obtain autologous lymphocytes.

**Fig. 1:**
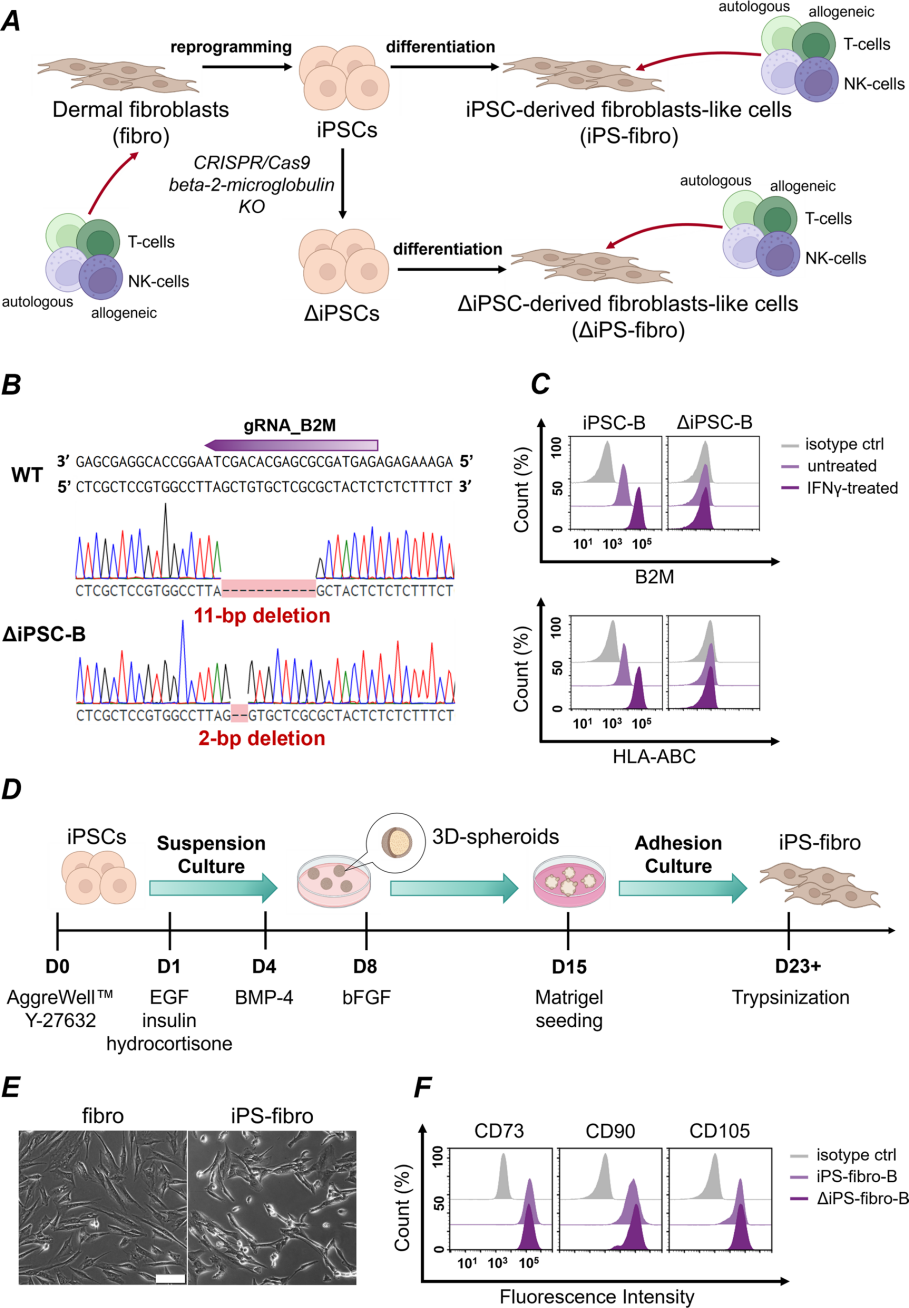
Construction of the isogenic cell model**.** **A** Schematic illustration of approaches used in this study. **B** Schematic illustration of gRNA target site in *B2M* gene and deletions in *B2M* alleles in ΔiPSC-B. **C** Flow cytometry analysis of B2M and HLA-ABC expression in iPSC-B and ΔiPSC-B with and without IFNγ-treatment. **D** Schematic diagram of the experimental protocol for iPS-fibro differentiation. **E** Morphology of fibroblasts and iPS-fibro (Scale bars, 50 μm). **H** Representative flow cytometry analysis of CD73, CD90, and CD105 (fibroblast markers) expression in iPS-fibro-B and ΔiPS-fibro-B

The scheme summarizing the approaches used in this study is illustrated in Fig. 1A. Here we report that cells differentiated from iPSCs provoked degranulation of allogeneic and autologous NK-cells regardless of HLA-I expression in target cells. In addition, we demonstrate that iPSC-derivatives had an improper balance of ligands for NK-cells receptors which led to the activation of NK-cells. IFNγ treatment tipped the balance and reduced NK-cell-mediated cytotoxicity toward iPSC-derived cells.

## Methods

### Cell culture

Human dermal fibroblasts and iPS-fibro were maintained in F3 medium containing DMEM medium (PanEco) supplemented with 10% FBS (Gibco), and 5 ng/ml bFGF (Miltenyi Biotec). The culture medium was replaced twice a week. A detailed description of fibroblast isolation procedure from skin biopsy is provided in Additional File 1.

Human iPSCs were generated from dermal fibroblasts of two healthy donors using the CytoTune™-iPS 2.0 Sendai Reprogramming Kit (ThermoFisher Scientific). The protocol for the generation of iPSCs is provided in Additional File 1. iPSCs were routinely maintained on Matrigel-coated (Corning) plates in Essential 8™ medium (Gibco). Cells were passaged with 0.05% Trypsin–EDTA solution (Gibco) and plated at 10–15% confluency in fresh Essential 8™ medium supplemented with 5 μM of ROCK inhibitor Y-27632 (Miltenyi Biotec). The culture medium was replaced daily.

### CRISPR/Cas9 gene editing of *B2M* gene

Guide RNA (gRNA) GAGTAGCGCGAGCACAGCTA targeting exon 1 of *B2M* gene was designed with CRISPOR [43] and cloned into a PX458 plasmid (Addgene #48138). iPSCs were transfected with TransIT^®^-LT1 Transfection Reagent (MirusBio) according to the manufacturer’s protocol. On day 2 after transfection, GFP-positive iPSCs were sorted by FACSMelody (BD Biosciences) and 2 × 10^4^ cells were seeded into Matrigel-coated 35 mm culture dishes in Essential 8™ + 10 μM Y27632 medium. On day 12 after transfection, single cell clones of potential *B2M* KO iPSCs were picked up manually and transferred to a 48-well plate for clonal expansion. On day 17 after transfection, we selected HLA-I negative iPSCs clones, and the lack of B2M and HLA-I expression was confirmed by flow cytometry. On-target genome editing of *B2M* gene was verified by Sanger sequencing. The fragments spanning the targeted region generated in PCR amplification were cloned into pAL2-T vector using Quick-TA kit (Evrogen). Recombinant plasmids from individual bacterial clones were then sequenced with M13 standard primers. Characterization of iPSCs was performed as described previously [44] with modest modifications (see Additional file 1).

### iPS-fibro differentiation from iPSCs

To differentiate iPSCs into iPS-fibro, we modified a previously reported protocol [45]. 3D-spheroids were formed from iPSCs suspension using AggreWell™800 24-well plates (STEMCELL Technologies Inc) according to the manufacturer's instructions. The next day, 3D spheroids were harvested into F1 medium containing 3/1 DMEM/F12 (Gibco), 5% FBS (Gibco), 0,1 μg/ml hydrocortisone (Gedeon Richter Ltd), 10 ng/ml EGF (Miltenyi Biotec) and 5 μg/ml insulin (Sigma). Further cultivation was carried out in special homemade mini-bioreactors [46] on an orbital shaker (70 rpm). From day 4, 2 ng/ml BMP-4 (R&D Systems) were added to the F1 medium. On day 8, the medium was shifted to the F3 medium. Onwards, the medium was changed every three days. On day 14, 3D spheroids were collected and plated on Matrigel-coated plates. In the next few days, 3D spheroids tightly adhered to the substrate, and the active migration of fibroblast-like derivatives was observed. From day 23, depending on the migration rate, iPS-fibro were dissociated with 0.25% Trypsin–EDTA solution (Gibco) and plated at the ratio of 1:4 in fresh F3 medium.

### Flow cytometry

Cells were harvested using Trypsin–EDTA (0.05 or 0.25%) to obtain single-cell suspensions. The staining and washing steps were performed in dPBS supplemented with 2% FBS. Cells were incubated with 100 μl diluted antibodies for 15 min at RT. After adding DAPI staining solution, the samples were analyzed on NovoCyte Flow Cytometer. All antibodies used in this study are listed in Additional file 1: Table S1.

### CD8^+^ T-cells and NK-cells isolation

PBMCs were isolated from buffy coats or whole blood using density gradient separation. CD8^+^ T-cells were isolated from fresh or thawed PBMCs using CD8 MicroBeads (Miltenyi Biotec) according to the manufacturer's protocol. NK-cells were sequentially isolated from the unlabeled cell fraction—CD8-depleted PBMC using NK-Cell Isolation Kit (Miltenyi Biotec). Lymphocytes were plated in X-VIVO 15 Medium (Lonza) at 2 mln/ml. A day before immunological tests, CD8^+^ T-cells and NK-cells were pre-activated using 500 U/ml IL-2 (Biotec).

### T-cell activation assay

75 × 10^3^ target cells were seeded into 48-well plates. All samples were set up in triplicate. The next day 10^5^ effector CD8^+^ T-cells were added to each sample well in 500 μl X-VIVO 15 medium supplemented with 4% heat-inactivated FBS and 100 U/ml IL-2. Cells were co-cultured for 7 days, then the percentage of CD69-expressing T-cells was measured by flow cytometry. Unstimulated CD8 + T-cells were used as a negative control. Day 7 for T-cell activation assay was chosen based on preliminary time-course experiment (Additional file 2: Fig. S1).

### NK-cell degranulation assay

5 × 10^4^ target cells at passages 4–7 (unless otherwise is specified) were seeded in flat-bottom 96-well plates. All samples were set up in triplicate. The next day 7 × 10^4^ NK-cells were added to each sample well in 70 μl X-VIVO 15 medium supplemented with antibodies against CD56 and LAMP-1 (CD107a). Immediately after NK-cells were added into the sample wells, the plates were centrifuged at 350 g for 5 min. Cells were co-cultured for 4 h, then the percentage of CD107a-expressing NK-cells was measured by flow cytometry. NK-cells co-cultured with cell line K562 was used as a reference positive control and unstimulated NK-cells were used as a negative control.

### NK-cell mediated cytotoxicity assay

2.5 × 10^4^ target cells at passages 4–6 were seeded in 96-well flat bottom plates. The next day NK-cells were added in 200 μl LDH medium at the indicated effector/target ratios and incubated for 4 h at 37 °C. Then 100 μl supernatants were collected and analyzed by Cytotoxicity Detection Kit (LDH) (Roche) following the manufacturer’s instructions. LDH medium (RPMI 1640 Medium, no phenol red (Gibco) supplemented with 1% HI-FBS) was used as background control. Target cells cultured alone, and unstimulated NK-cells were used as controls for spontaneous LDH release. Lysed with 1% Triton-100 (Ferak Berlin) target cells at endpoint were used as maximum LDH release. All group samples were set up in triplicate. NK-cell cytotoxicity was calculated as follows:

$${\text{cytotoxicity}}, \% = \frac{{\left[ {{\text{effector:}}\;{\text{target}}\;{\text{cell}}\;{\text{mix}}} \right] - \left[ {{\text{effector}}\;{\text{cell}}\;{\text{control}}} \right] - [{\text{target}}\;{\text{spontaneous }}\;{\text{release}}]}}{{\left[ {{\text{target}}\;{\text{maximum }}\;{\text{release}}} \right] - [{\text{target }}\;{\text{spontaneous}}\;{\text{release}}]}}$$

### RNA isolation and NGS library preparation

Total RNA was isolated using RNeasy Mini Kit (Qiagen) according to the manufacturer’s instructions. The on-column DNase treatment was performed with RNase-Free DNase Set (Qiagen). RNA quality was validated using 2100 bioanalyzer (Agilent Technologies). Enrichment of polyadenylated RNA and library preparation was performed with NEBNext Ultra II Directional RNA Library Prep Kit (NEB) according to the manufacturer’s protocol. The library underwent a final cleanup using the Agencourt AMPure XP system (Beckman Coulter) after which the libraries’ size distribution and quality were assessed using a high sensitivity DNA chip (Agilent Technologies). Libraries were subsequently quantified by Quant-iT DNA Assay Kit, High Sensitivity (ThermoFisher). Samples were sequenced on NextSeq 500 System (Illumina) with NextSeq 500/550 High Output Kit v2.5 (75 Cycles).

### Analysis of RNA-seq data

For all samples, raw sequencing data was trimmed for the adaptor sequence and quality control with “Trim Galore” (v.0.5.0). Trimmed RNAseq reads were aligned against the Homo Sapiens GRCh38.13 genome annotation at the transcript level using “Salmon” software (v. 1.4). Next, results were aggregated to gene level using the “R” package “tximport.” Datasets were filtered to remove rows with only a single count across all samples and differentially expressed genes were identified using the “R” package “DESeq2.” “R” packages “FactoMineR” and “ggplot2” were used for PCA analysis and data visualization, respectively. For functional annotation of differentially expressed genes, we used the “topGo” package in “R.” A *p*-value correction for multiple testing was made using an FDR method, and the cut-off threshold was 0.05. Also, we performed GSEA enrichment analysis using “msigdbr” and “clusterprofiler” packages in “R” against the hallmark pathway gene set from the Molecular Signatures Database (MSigDB). The raw sequence data and processed data have been submitted to NCBI Gene Expression Omnibus (GEO) data repository with accession number GSE212796.

### Analysis of public RNA-seq data

Published unprocessed RNA-Seq samples of fibroblasts reprogrammed to iPSCs, iPSCs, and iPSC-derived fibroblast-like cells were downloaded from the NCBI GEO data repository (accession numbers GSE61390, GSE62772, GSE73211). Gene expression levels and differential expressed genes for each dataset were calculated in the same way as the RNA-seq data we derived. Pearson correlation coefficients were calculated from DESeq2 normalized counts and plotted using the “corrplot” package in “R.” The relationship between samples was also assessed by principal component analysis of DESeq2 rlog-transformed counts using “R” package “FactoMineR” and the expression heatmap was generated using “R” package “pheatmap.” The overlap between differentially expressed genes identified from all datasets has been estimated using “R” package “UpSetR.”

### Real-time RT-PCR

cDNA synthesis was performed using MMLV RT-kit (Evrogen). qPCR was performed using qPCRmix-HS SYBR (Evrogen) with gene-specific primers (Additional file 1: Table S2) in CFX96 Touch Real-Time PCR Detection System (Bio-Rad). mRNA expression was normalized to the housekeeping gene GAPDH. All group samples were set up in triplicate.

### Statistical analysis

Statistical analysis was performed with Prism 9.3.1 software (GraphPad). All data shown in the study are presented as mean ± S.E.M. Statistical significance was determined by ordinary one-way, repeated measures (RM) one-way or two-way ANOVA tests followed by Tukey’s multiple comparison test. Differences were considered significant at *p* < 0.05.

## Results

### Generation of iPS-fibro and ΔiPS-fibro

Dermal fibroblasts of donors A and B (48- and 26-years old females, respectively) were reprogrammed into iPSCs using the delivery of Yamanaka’s factors by integration-free Sendai virus. We obtained the iPSCs cell lines IPSFF1S and IPSFD4S for donors A and B, respectively. In this paper, we use designations according to the donor: iPSC-A and iPSC-B. The iPSC-A and iPSC-B were characterized according to standard criteria [20, 47]. Using CRISPR/Cas9 genome editing for the *B2M* gene knockout, we obtained ΔiPSC-A and ΔiPSC-B lines lacking HLA-I expression. A detailed description of ΔiPSC-A was previously published [20]. The iPSC-A and its subclone ΔiPSC-A are registered in hPSCreg database [48]. The ΔiPSC-B was derived upon the same procedure described briefly below.

For CRISPR-Cas9 genome editing, we used the PX458 vector [49] with a gRNA targeting the first exon of the *B2M* gene, which encodes a signal peptide of the B2M protein. A schematic illustration of the gRNA-targeted sequence of the human *B2M* gene is shown in Fig. 1B. After the cell sorting, selected clones were analyzed for B2M and HLA-I expression by flow cytometry. Expression of B2M and HLA-ABC was not detected in ΔiPSC-B even after IFNγ treatment, confirming that the functional knockout of the *B2M* gene led to a complete deficiency of HLA-I proteins on the cell surface (Fig. 1C). In addition, *B2M* knockout in ΔiPSC-B was validated by Sanger sequencing. We revealed deletions of 11 bp and 2 bp in alleles of the *B2M* gene in ΔiPSC-B, both causing a frameshift mutation (Fig. 1B). ΔiPSC-B displayed typical pluripotent stem cell morphology and maintained normal karyotype 46, XX (Additional file 2: Fig. S2D). They expressed key markers for pluripotency in nuclei (OCT4, SOX2, NANOG) and cell surface (SSEA-4, TRA-1–60) (Additional file 2: Figs. S2A, S2C, S3). Upon spontaneous in vitro differentiation, ΔiPSC-B derivatives displayed markers of all three germ layers, i.e., ectoderm (CK18), mesoderm (CD31), and endoderm (HNF4A) (Additional file 2: Fig. S2B). Thus, ΔiPSC-B maintained pluripotency after genome editing.

The wild-type iPSCs and ΔiPSCs were differentiated into fibroblast-like cells (iPS-fibro and ΔiPS-fibro) through the stage of 3D spheroids. The main inductors of differentiation were EGF, BPM-4 and bFGF. The differentiation protocol is shown in Fig. 1D. In brief, 3D spheroids formed from iPSCs were cultured in a dynamic suspension for 14 days. They were then transferred to Matrigel-coated plates, where cells migrated, forming a monolayer. The differentiated cells had fibroblast-like morphology (Fig. 1E) and expressed markers specific for fibroblasts: CD73 (ecto-5'-nucleotidase), CD90 (Thy-1), and CD105 (endoglin) (Fig. 1F). Neither B2M nor HLA-ABC expression was detected in ΔiPS-fibro-A and ΔiPS-fibro- B (Additional file 2: Fig. S2E).

### iPSC-derivatives did not elicit increased T-cell responses compared with somatic cells

**Fig. 2:**
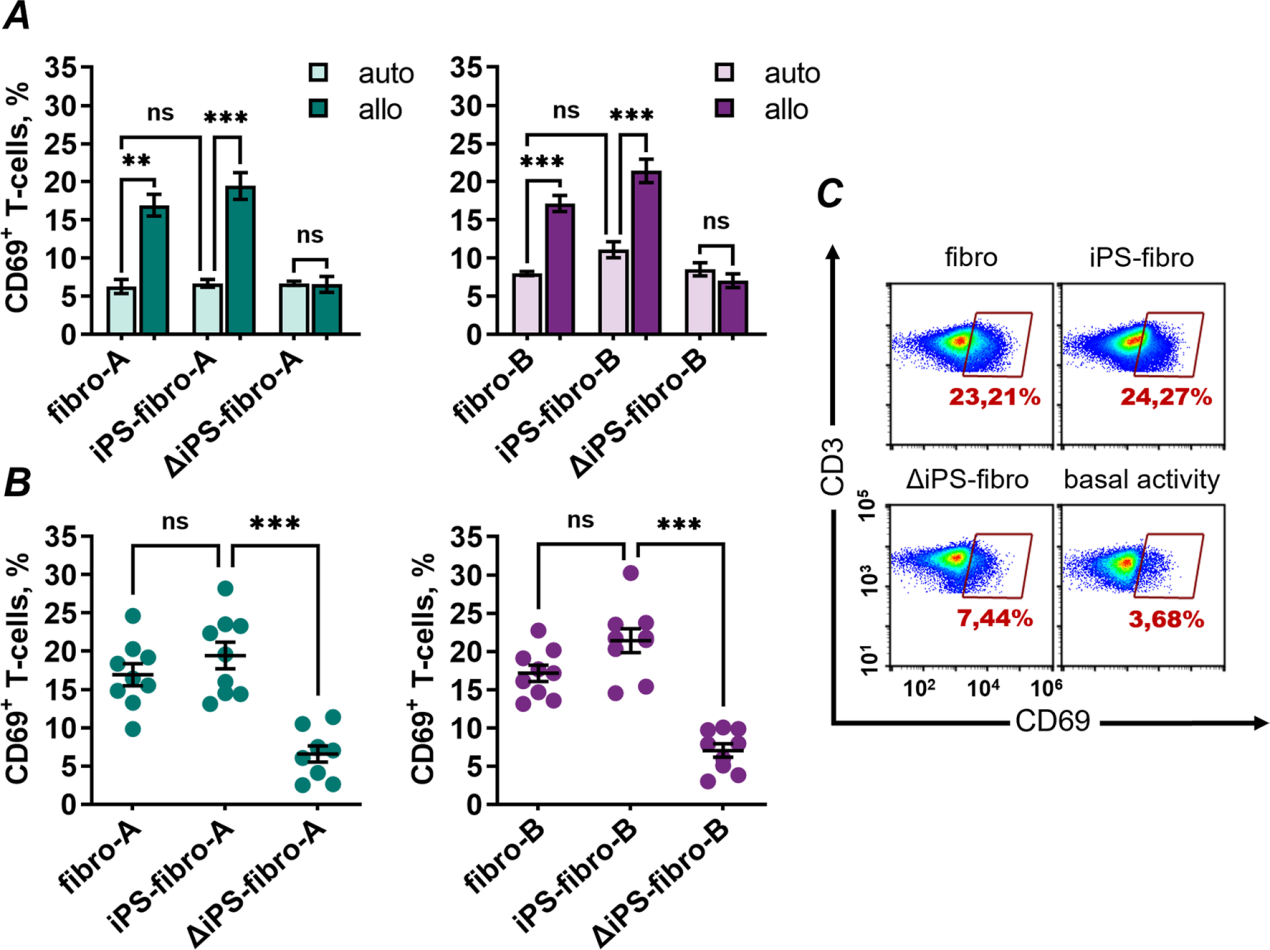
In vitro T-cell activity against dermal fibroblasts and isogenic iPSC-derivatives. **A** The allogenic T-cells (*N* = 9) co-cultured with isogenic dermal fibroblasts or iPS-fibro demonstrated increased expression of activation marker CD69 compared to autologous T-lymphocytes. The bars represent the mean ± SEM. **P < 0.01; ***P < 0.001; two-way ANOVA. **B** Number of allogenic CD69^+^ T-cells (*N* = 9) was greatly reduced against ΔiPS-fibro. The bars represent the mean ± SEM. The dots represent independent experiments with each donor's T-cells performed in triplicates. ***P < 0.001; RM one-way ANOVA. **C** Representative flow-cytometry plots illustrate CD69 expression for T-lymphocytes of an allogenic donor. T-cells cultured without target cell were used as negative control

First, we compared allogeneic and autologous T-cell responses promoted by isogenic dermal fibroblasts and iPS-fibro of donors A and B. Upon co-cultivation, a 2.5 times higher percentage of allogeneic compared to autologous T-lymphocytes upregulated surface CD69, regardless of whether primary fibroblasts or iPS-fibro were used as targets (Fig. 2A). A similar T-cell activation level against fibroblasts and iPS-fibro was also observed for autologous T-cells (Fig. 2A). These results indicate that possible immunopeptidome discrepancies caused by reprogramming and culture procedures did not elicit broad memory T-cell responses in both autologous and allogenic models.

In addition, we showed that the absence of foreign HLA class I molecules reduced the activation of allogeneic T-cells against ΔiPS-fibro (Fig. 2B). Meanwhile, as we expected, the absence of “self” HLA class I molecules did not affect the immune response of autologous T-lymphocytes (Fig. 2A). Moreover, for ΔiPS-fibro, we did not observe a difference in the activation of autologous or allogeneic effector cells (Fig. 2A). This data proves that manipulating HLA expression in hPSCs can lead to the immunological tolerance of hPSC-derivatives to allogeneic T-cells.

### iPSC-derivatives were vulnerable to NK-cell degranulation and cytotoxicity regardless of HLA-I status

Next, we examined NK-cell immune responses to isogenic dermal fibroblasts and iPSC-derivatives. In the analysis of T-cell activity, we observed low variation among donors in the expression of the CD69 activation marker (Fig. 2B). The low variation allowed for the direct comparison of CD69^+^ T-cells activated by co-culturing with analyzed cells. On the contrary, in the CD107a mobilization assay, we observed substantial variation among different donors in the number of degranulated NK-cells (Additional file 2: Fig. S4A). Furthermore, considerable variation was also observed in independent experiments with NK-cells from the same donor (Additional file 2: Fig. S4B). Therefore, we introduced the degranulation index calculated as a ratio of the number of CD107a^+^ NK-cells to the number of CD107a^+^ NK-cells co-cultured with the positive control’s K562 cells:

$${\text{Degranulation }}\;{\text{index = }}\frac{{{\text{CD107a}}^{ + } \;{\text{NK }}\;{\text{cells }}\;{\text{in }}\;{\text{analyzed }}\;{\text{samples}}}}{{{\text{CD107a}}^{ + } \;{\text{NK}}\;{\text{ cells }}\;{\text{in }}\;{\text{positive }}\;{\text{control}}}}$$

**Fig. 3:**
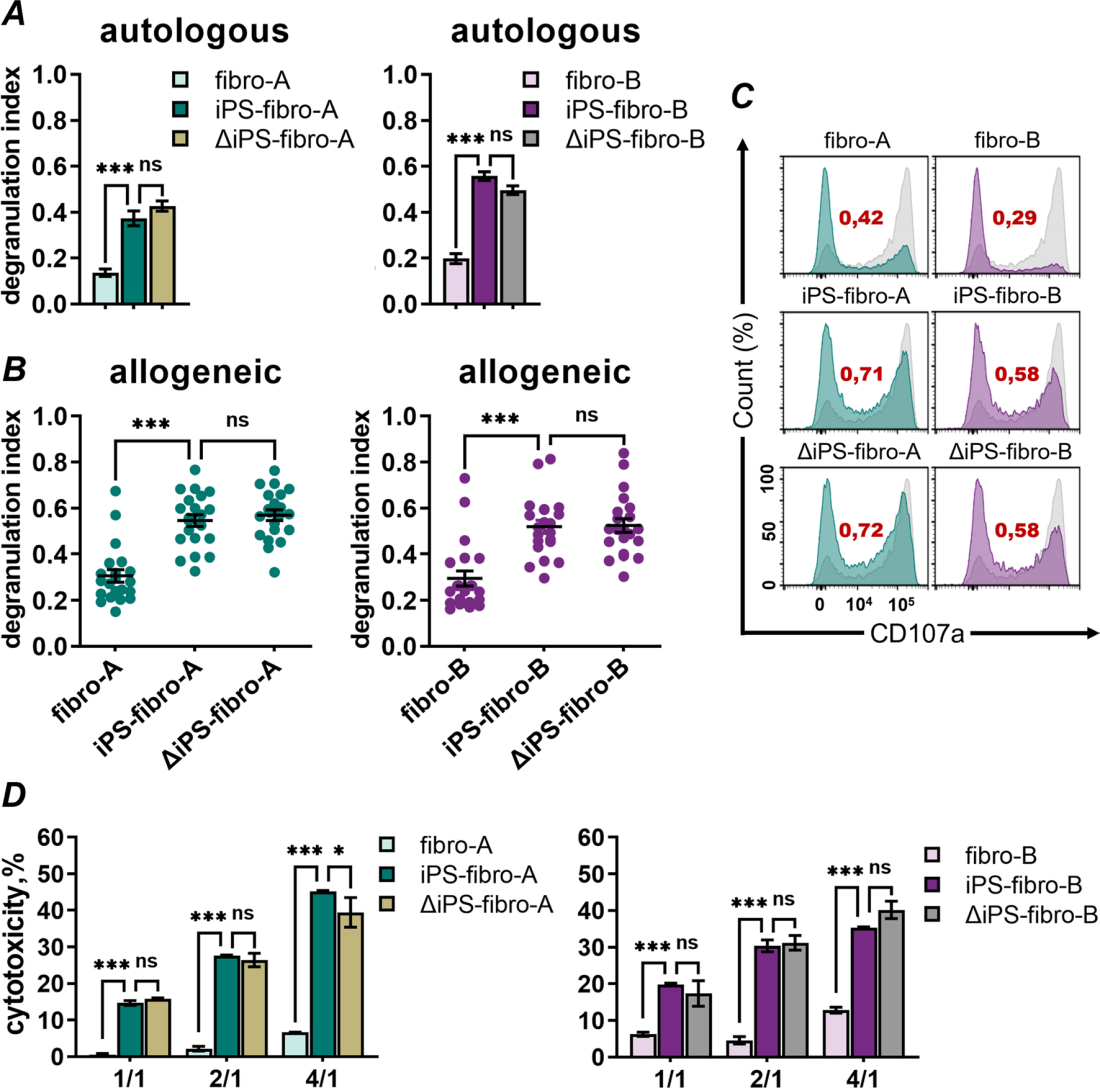
In vitro NK-cell response to dermal fibroblasts and isogenic iPSC-derivatives. **A** Autologous NK-cells demonstrated significantly higher degranulation against iPS-fibro and ΔiPS-fibro compared to dermal fibroblasts. The bars represent the mean ± SEM. ***P < 0.001; ordinary one-way ANOVA. **B** The degranulation index of allogenic NK-cells (*N* = 21) did not differ between iPS-fibro and ΔiPS-fibro. Each dot represents an independent experiment performed in triplicates. The dots represent the mean ± SEM. ***P < 0.001; RM one-way ANOVA. **C** Representative flow-cytometry histograms illustrate CD107a expression for NK-cells of an allogenic donor co-cultured with dermal fibroblasts and isogenic iPSC-derivatives. Degranulation against the K562 cells used as the positive control is indicated in gray. The estimates of the degranulation index are given in red. **D** The LDH release assay demonstrated NK-cell cytotoxicity against dermal fibroblasts and isogenic iPSC-derivatives. The bars represent the data on cytotoxicity of an allogenic donor's NK-cells mixed at the different effector/target (E/T) ratios. Each bar represents an independent experiment performed in triplicates. The bars represent the mean ± SEM. *P < 0.05; **P < 0.01; ***P < 0.001; two-way ANOVA

According to the “missing-self” hypothesis, one of the major functions of NK-cells is the recognition of cells lacking self-HLA class I molecules. As expected, autologous (Fig. 3A) and allogeneic (Fig. 3B) NK-cell activity against ΔiPS-fibro lacking HLA-I was high. Surprisingly, we observed that wild-type iPSC-derivatives provoked the response of both autologous (Fig. 3A) and allogenic (Fig. 3B) NK-cells to the same extent as isogenic ΔiPS-fibro. Meanwhile, we observed lower NK-cell activity against parental fibroblasts compared to iPS-fibro (Fig. 3A). The degranulation index of allogeneic NK-cells against iPS-fibro was 1.7 times higher compared to that of isogenic dermal fibroblasts. That difference was even higher for autologous NK-cells. The autologous NK-cell response to iPS-fibro was 2.7 times higher compared to isogenic dermal fibroblasts (Fig. 3A). CD107a mobilization assay data was consistent with LDH cytotoxicity tests. On all effector/target ratios, the level of NK-cell cytotoxicity was higher against iPSC-derivatives but not against dermal fibroblasts (Fig. 3D). We also tested NK-cell response to other iPSC-derived cells: retinal pigment epithelium (iPS-RPE) and cardiomyocytes (iPS-CM). These cells were also susceptible to the cytotoxic properties of NK-cells (Additional file 2: Fig. S4F, S4I). The differentiation protocols as well as characteristics of iPSC-derived RPE and CM are presented in Additional files 1 and 2: Fig. S4C–E and S4G–H.

### Gene expression profiling revealed the imbalance of NK-cell ligands in iPSC-derivatives

The NK-cell can respond to increased signals from activating receptors and decreased signals from inhibitory receptors. In this regard, manipulation of signal intensity coming from activating NK-cell receptors should reduce immune responses toward target cells. We demonstrated that blocking some activating NK-cell receptors, such as NKG2D and DNAM-1, mitigated NK-cell degranulation against both wild-type and *B2M* KO iPSC-derived cells (Additional file 2: Fig. S5A and S5B). Therefore, we assumed that an improper balance of NK-cell ligands in iPSC-derivatives was responsible for provoking an intensive NK-cell response. To test this, we performed gene expression profiling in isogenic dermal fibroblasts, iPS-fibro, and ΔiPS-fibro from donors A and B. To increase the resolution of the analysis, we included analogous samples from donor C. Our main goal was to identify differentially expressed genes encoding ligands for activating and inhibitory NK-cell receptors.

**Fig. 4:**
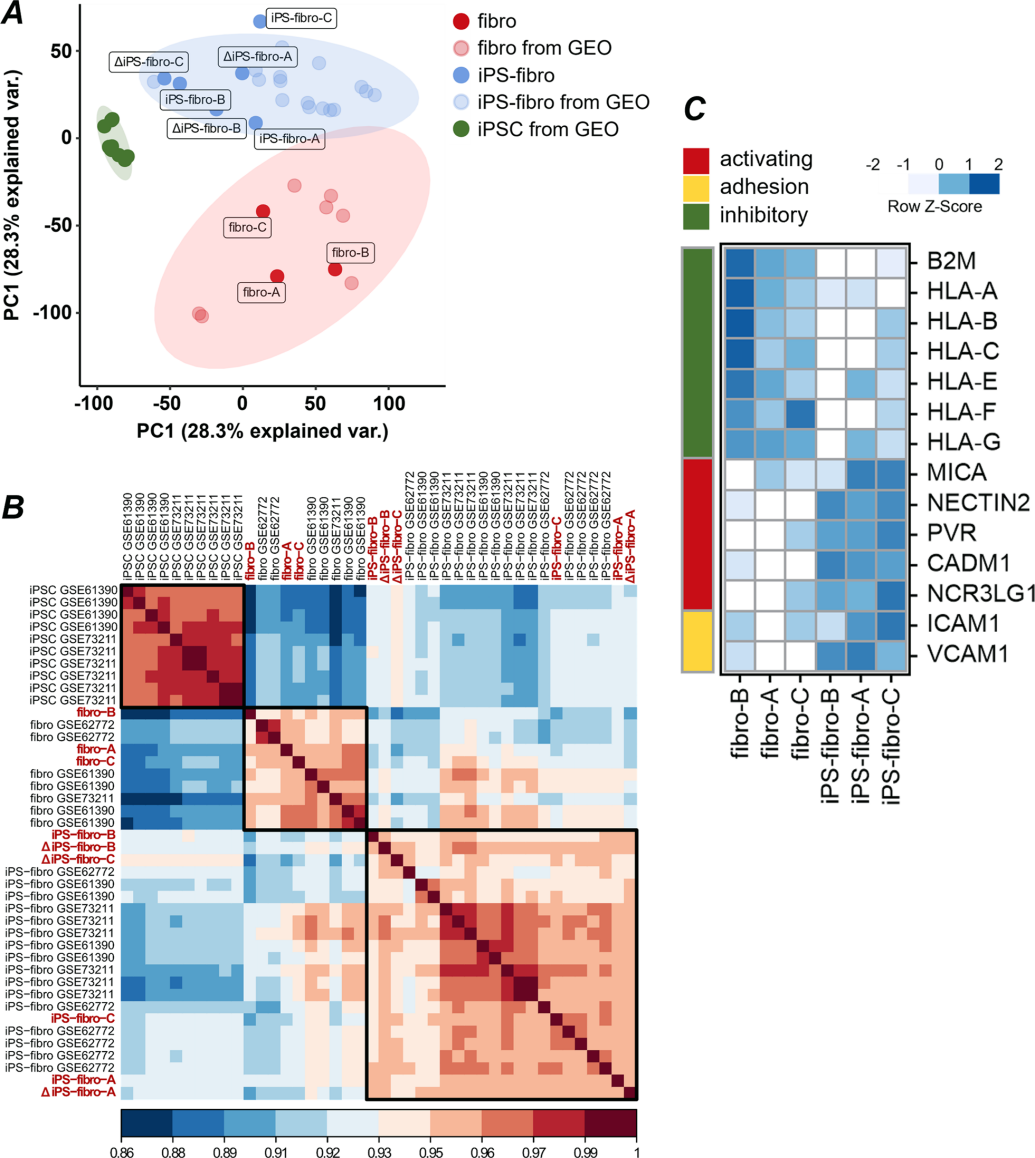
Gene expression profiling of dermal fibroblasts and isogenic iPSC-derivatives. **A** The PCA plots clearly separates 3 types of samples: undifferentiated iPSCs, parental fibroblasts, and iPSC-derived fibroblast-like cells (iPS-fibro). **B** The correlation matrix demonstrating the high degree of similarity between human fibroblasts and iPS-fibro. Samples obtained in this study are indicated in red. **C** Transcriptome profiles were used to analyze expression of activating and inhibitory NK-cell ligands, as well as adhesion molecules in dermal fibroblasts and iPS-fibro. The expression values of each gene in a row are normalized by a row Z-Score

We first assessed whether iPS-fibro derivatives were similar to primary human fibroblasts. Using third-party scRNA-seq data [50], we identified 22 key markers typical for fibroblasts and 29 markers typical for undifferentiated iPSCs (Additional File 2: Fig. S6A). Next, we selected three publicly available datasets consisting of the transcriptome data (GSE61390, GSE62772, GSE73211) on fibroblasts reprogrammed to iPSCs, iPSCs, and iPSC-derived fibroblast-like cells [51–53]. The analysis of RNA-sequencing data (Fig. 4A) demonstrated that dermal fibroblasts and iPS-fibro expressed key fibroblasts markers, whereas iPSCs were hierarchically clustered by key markers typical for undifferentiated cells (Additional File 2: Fig. S6B). Despite a correlation over 0.9 between our iPSC-derivatives and human fibroblasts (Fig. 4B), the highest level of correlation (~ 0.95) was observed between our iPS-fibro and independently obtained iPSC-derived fibroblast-like cells (Fig. 4B). These results indicate that the transcriptomic signature of fibroblast-like iPSC-derivatives was reproducible across laboratories regardless of differences in reprogramming, differentiation, and cultivation.

We then identified differentially expressed genes in our iPS-fibro compared to parental fibroblasts (Additional file 2: Fig. S7A). Transcriptomic analysis revealed that 1670 genes were downregulated. Among them were 13 genes encoding ligands for NK-cell receptors or other molecules necessary for NK-cell activation (GO:0030101) (Additional file 3). Similarly, 1597 genes were upregulated in iPS-fibro, with 12 of them encoding molecules necessary for NK-cell activation. Gene Ontology enrichment analysis showed that genes downregulated in iPS-fibro were significantly enriched in several immunological pathways, including immune response, immune effector response, and inflammatory response (Additional file 2: Fig. S7C). Gene Set Enrichment Analysis revealed that genes from sets “Hallmark interferon-gamma response” and “Hallmark interferon-alpha response” were significantly downregulated in iPS-fibro (Additional file 2: Fig. S7E).

Next, we focused on the pattern of NK-cell ligands expression (Fig. 4C; Additional file 2: Fig. S7D). We found that many genes encoding ligands for inhibitory and activating receptors were differentially expressed in iPSC-derivatives compared with primary fibroblasts. Alongside this, there was a consistency in the expression of ligands for NK-cell receptors between iPS-fibro and ΔiPS-fibro (Additional file 2: Fig. S7B).

HLA class I molecules serve as ligands for two main classes of inhibitory NK-cell receptors: the KIR (Killer-cell immunoglobulin-like receptors) family and the CD94-NKG2A heterodimer. This interaction underlies the molecular basis of “missing-self” recognition. Compared to their parental fibroblasts, the expression of all classical HLA class I transcripts (*HLA-A*, *HLA-B*, and *HLA-C*) was downregulated in iPS-fibro with fold change > 2. Likewise, the expression of the light chain of HLA class I molecules, *B2M*, was also downregulated in iPS-fibro. The downregulation of *HLA-A*, *HLA-B*, *HLA-C*, and *B2M* genes for iPS-fibro-A and iPS-fibro-B was validated by RT-qPCR (Additional file 2: Fig. S8A). The difference between fibro-B and iPS-fibro-B was more pronounced than in other isogenic systems. This phenomenon may be explained by the comparatively higher expression of HLA-I molecules for fibro-B and might be a donor-specific feature.

The level of non-classical HLA class I expression was also reduced in iPS-fibro. Non-classical HLA-I includes *HLA-E*, *HLA-G*, and *HLA-F* genes that exert immunomodulatory properties in NK-cells. Accordingly, the decline in their expression might also promote the manifestation of NK-cell cytotoxic functions. On the other hand, no differentially expressed genes were identified among the other ligands for minor inhibitory receptors (such as PD-1, NKRP1A, CEACAM1, CD96, TIGIT, KLRG1, and TIM-3). Hence, we assumed that a relatively low level of HLA class I molecules in the iPS-fibro led to the deficiency in inhibitory signals that might tip the balance toward the activation of the cytotoxic program of NK-cells.

Almost half of the genes encoding the key ligands for activating NK-cell receptors were differentially expressed in iPS-fibro (Fig. 4C). Upregulated genes included the genes of ligands for dominant activating receptors: NKG2D, DNAM-1, and natural cytotoxicity receptors (NCRs). Compared to their parental fibroblasts, the stress-induced molecule’s *MICA* (NKG2D ligand) gene expression was more than 1.5 times higher in iPS-fibro. The DNAM-1 ligands, *NECTIN2* (CD112) and *PVR* (CD155), and the NKp30 ligand, *NCR3LG1* (B7-H6), underwent a more noticeable increase in gene expression with fold-change of > 3 in iPS-fibro. The upregulation of *MICA*, *ULBP3*, *NECTIN2*, and *PVR* genes for iPS-fibro-A and iPS-fibro-B was validated by RT-qPCR (Additional file 2: Fig. S8B). Finally, some genes such as *CADM1* (CRTAM ligand) and *CD70* (CD27 ligand) were expressed only in iPSC-derivatives but not in parental fibroblasts. Notably, an imbalance in the expression of ligands for activating NK-cell receptors was also observed in publicly available RNA-seq datasets. In particular, *NECTIN2*, *PVR*, *CADM1,* and *CD70 gene* expression was upregulated in independently derived fibroblast-like cells (data not shown). Presumably, the upregulation of these genes might result from the incomplete maturation of fibroblast-like iPSC-derivatives.

In addition, we analyzed the expression of genes encoding adhesion molecules. The interaction of adhesion molecules with their receptors on NK-cells contributes to firming NK-cell adhesion to the target cell and leads to the assembly of immunological synapse essential for target cell killing [54]. *ICAM-1* (LFA-1 ligand) and *VCAM-1* (VLA-4 or integrin α4β1 ligand) genes were upregulated in iPSC-derivatives (Fig. 4C). The same change was observed in publicly available RNA-seq datasets (data not shown). The overexpression of some adhesion molecules might also contribute to NK-cell mediated cytotoxicity against iPSC-derivatives.

Various factors have affected the balance regulating the response of NK-cells to iPS-fibro. First, we observed a relatively low gene expression of HLA-I molecules, major inhibitory ligands, in iPS-fibro. Second, genes coding for main activating NK-cell ligands were upregulated in iPS-fibro. Third, the genes of some adhesion molecules were also overexpressed in iPS-fibro.

### Prolonged iPS-fibro cultivation promoted HLA-I upregulation but failed to inhibit NK-cell response

**Fig. 5:**
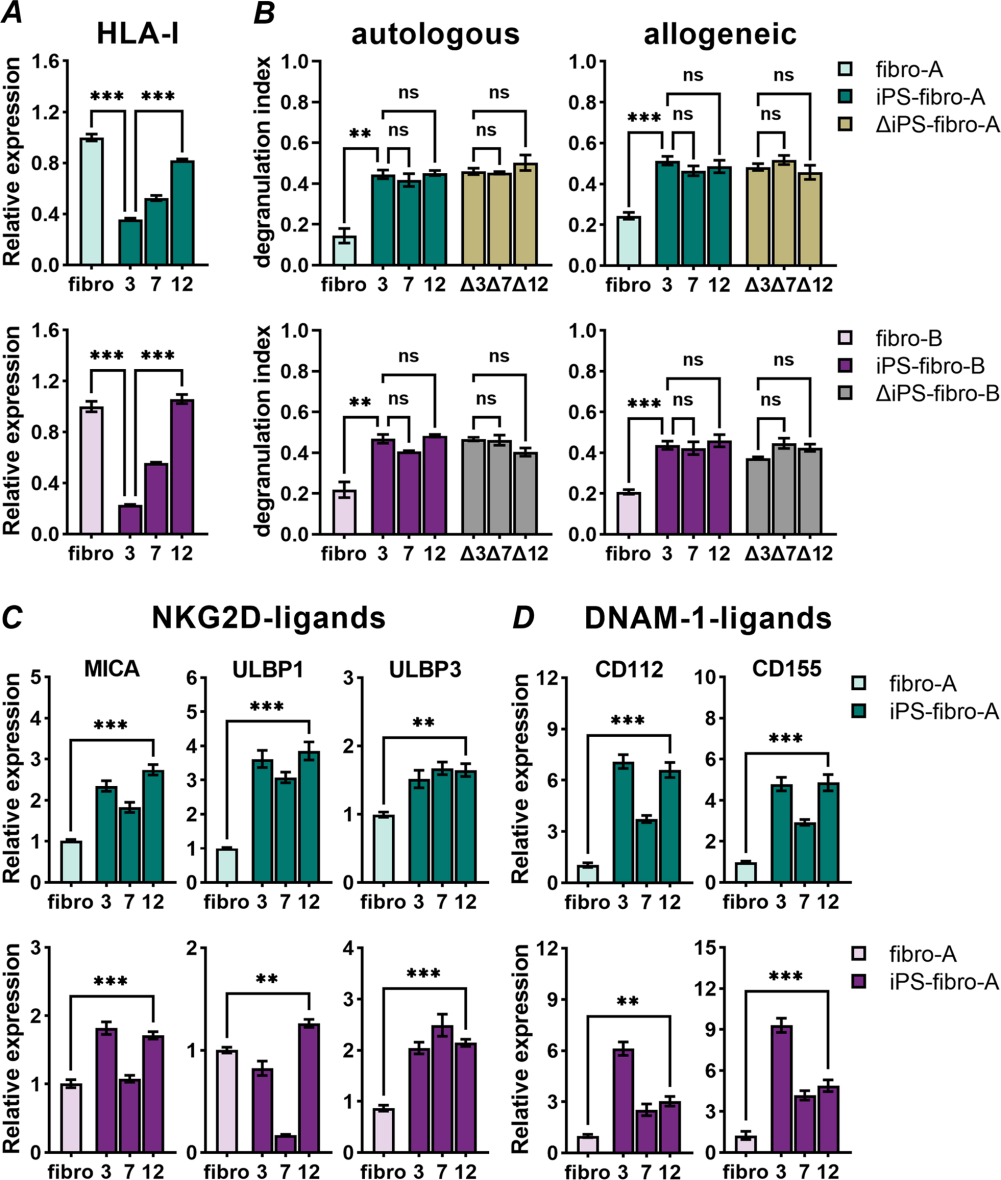
Prolonged iPS-fibro cultivation promoted HLA-I upregulation but failed to inhibit NK-cell responses. **A** A gradual increase of HLA-ABC expression was observed during the process of passaging and maturation of iPS-fibro. HLA-ABC expression was normalized to isogeneic parental fibroblasts. The bars represent the mean ± SEM; ***P < 0.001; ordinary one-way ANOVA. **B** Prolonged passaging of iPS-fibro had no effect on the immune response mediated by autologous or allogeneic (*N* = 9) NK-cells. Each bar represents the mean ± SEM; **P < 0.01; ***P < 0.001; RM one-way ANOVA. **C** Expression of ligands for NKG2D activating receptor in parental fibroblasts and iPS-fibro at different passages. Expression of all analyzed genes in iPS-fibro was normalized to isogeneic parental fibroblasts. The bars represent the mean ± SEM; **P < 0.01; ***P < 0.001; RM one-way ANOVA. **D** Expression of ligands for DNAM-1 activating receptor in parental fibroblasts and iPS-fibro at different passages. Expression of all analyzed genes in iPS-fibro was normalized to isogeneic parental fibroblasts. The bars represent the mean ± SEM; **P < 0.01; ***P < 0.001; RM one-way ANOVA

Since the low expression of HLA class I molecules is a common feature of hPSCs, the low expression of HLA class I molecules by intact iPS-fibro might be associated with immaturity. Therefore, we evaluated the HLA-ABC and B2M expression by parental fibroblasts and iPS-fibro at different passages. We showed that the most significant difference in the expression of HLA-ABC and B2M was observed between parental fibroblasts and iPS-fibro at passage 3, i.e., the “youngest” iPSC-derived cells (Fig. 5A; Additional file 2: Fig. S9A). Further in the process of cultivation and passaging, a significant increase in the HLA-ABC and B2M expression was observed (Fig. 5A; Additional file 2: Fig. S9A). However, it was not sufficient to alter NK-cell immune response both in autologous and allogeneic models (Fig. 5B). The degranulation index against iPSC-derivatives did not change over the course of passaging even when iPS-fibro-B at passage 12 reached the fibroblast-specific level of HLA-I expression (Fig. 5A, bottom panel). Next, we analyzed the pattern of activating NK-cell ligands in iPS-fibro at different passages. We found that more mature iPS-fibro maintained elevated gene expression of ligands for NKG2D (Fig. 5C) and DNAM-1 (Fig. 5D) NK-cell receptors. Thus, prolonged culture and passaging of iPS-fibro failed to reach the equilibrium state of NK-cell ligands. The upregulation of HLA-I expression in iPS-fibro at passage 12 was insufficient to counterbalance the predominance of activating signals, resulting in increased NK-cell response similar to "young" iPSC-derived cells (Fig. 5B).

### IFNγ treatment increased HLA-I expression and reduced NK-cell-mediated cytotoxicity toward iPS-fibro

The elevated expression of ligands for NKG2D (Fig. 5C) and DNAM-1 (Fig. 5B) receptors led to a decisive advantage of activating NK-cells against iPS-fibro at all passages. Therefore, it is necessary to boost the HLA class I expression level to bring the balance of activating and inhibitory ligands into an equilibrium state. A considerable shift in the balance toward inhibition should lead to a decrease in NK-cell activation and cytotoxicity. Therefore, we analyzed whether changing the proportion of activating and inhibitory ligands for NK-cell receptors in iPS-fibro was possible. For this purpose, we used the important proinflammatory cytokine IFNγ that was previously shown as a potent inducer of HLA class I expression [55].

**Fig. 6:**
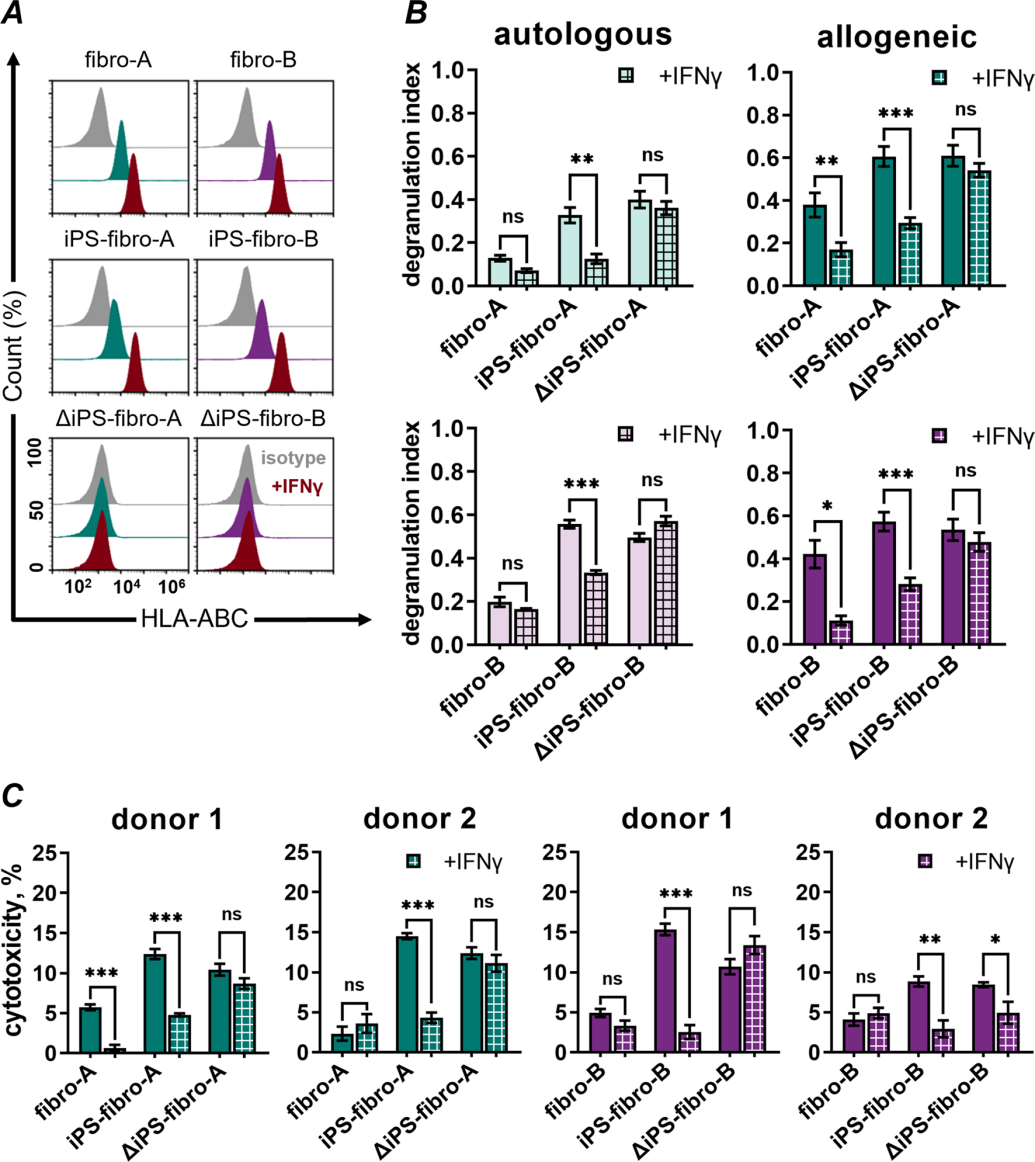
The pretreatment of iPS-fibro with IFNγ reduced the level of NK-cell degranulation and cytotoxicity*.* **A** Flow cytometry analysis revealed an increase in HLA-ABC expression in fibroblasts and iPS-fibro after IFNγ-stimulation (indicated in red). Isotype control is indicated in gray. **B** The autologous and allogeneic (*N* = 7) NK-cells demonstrated significantly reduced degranulation against iPS-fibro pretreated with IFNγ. Each bar represents the mean ± SEM; *P < 0.05; **P < 0.01; ***P < 0.001; RM one-way ANOVA. **C** The LDH release assay demonstrated a reduction in NK-cell cytotoxicity against iPS-fibro pretreated with IFNγ. The bars represent data on the cytotoxicity of two allogenic donors’ NK-cells. Each bar represents an independent experiment performed in triplicates. The bars represent the mean ± SEM; *P < 0.05; **P < 0.01; ***P < 0.001; ordinary one-way ANOVA

As expected, the IFNγ treatment greatly enhanced the HLA-I gene and protein expression. Twofold increase was observed for dermal fibroblasts, and more than sixfold increase was observed for iPS-fibro (Fig. 6A; Additional file 2: Fig. S10A). Similarly, a more than fivefold upregulation of the *B2M* gene and protein was detected after IFNγ stimulation (Additional file 2: Fig. S10A). It is worth noting that after the pretreatment with IFNγ, HLA-I expression in iPS-fibro was 3.5 times higher than in intact parental fibroblasts (Additional file 2: Fig. S9B). Moreover, IFNγ also increased the expression of minor inhibitory NK-cell ligands, such as *CEACAM1* and *LGALS9*, and other molecules that can also have inhibitory effect on NK cells, such as *CD274* (PD-L1) [56] and *IDO* [57] (Additional file 2: Fig. S10B, S10C). At the same time, IFNγ treatment affected the gene expression of some activating NK-cell ligands such as *MICB* and *ICAM-1* (Additional file 2: S10C).

Next, we assessed whether IFNγ stimulation could alter the NK-cell response. The response of autologous and allogenic NK-cells declined by about half toward IFNγ-pretreated iPS-fibro compared to their unstimulated counterparts (Fig. 6B). Interestingly, the NK-cell response to IFNγ-pretreated iPS-fibro dropped nearly to values typical for intact isogeneic fibroblasts. In contrast, IFNγ-stimulation did not alter NK-cell responses to ΔiPS-fibro with the knockout of the *B2M* gene. Both the degranulation index and NK-cell cytotoxicity remained at a level comparable to untreated samples (Fig. 6B, C). NK-cell activation against autologous fibroblasts also changed only slightly. Meanwhile, allogeneic NK-cells significantly reduced the release of cytotoxic granules after IFNγ treatment of parental fibroblasts (Fig. 6B). Most likely, such a considerable difference with stimulated fibroblasts was due to an excessive response to unstimulated cells by hyperactivated NK-cell of some donors (Fig. 3B). Contrariwise, there was no mitigation in cytotoxicity against parental fibroblasts pretreated with IFNγ. The reduced cytotoxic effect was observed only when IFNγ-pretreated fibro-A were co-cultured with NK-cells of donor 1 (Fig. 6C).

Summarizing the results of IFNγ treatment, we can conclude that weak change in activating ligands and considerable boost in the expression of HLA-I and other inhibitory molecules provided the predominance of inhibitory signals in IFNγ-stimulated iPS-fibro. This, in turn, normalized NK-cell response toward iPS-derived cells.

## Discussion

Autologous iPSC-differentiated derivatives are a promising technology for regenerative medicine, which critically depends on donor tissues and organs [58]. Autologous grafts of iPSCs origin are considered immunotolerant [38, 59]. Therefore, patients transplanted with autological iPSC-derivatives might avoid immunosuppressive therapy, which has many side effects [11]. However, we report here that iPSC-derivatives could be immunologically intolerant because they triggered the activation and cytotoxicity of autologous lymphocytes. Specifically, we observed that autological NK-cells responded 2.5 times more to iPSC-derivatives than to their parental somatic cells used for iPSCs reprogramming. We observed this phenomenon in 3 types of iPSC-derived somatic cells, namely fibroblast-like cells, cardiomyocytes, and RPE cells.

Previously, some groups reported a T-cell response to syngeneic and autologous iPSC-derived cells [39, 40]. However, other studies contradicted the above-mentioned results and demonstrated a negligible immunogenicity of differentiated syngeneic iPSC-derivatives [60, 61]. In this study, we did not observe an enhancement of T-cell response to iPS-fibro compared to the parental somatic cells. The main role of T-cells is the recognition of foreign molecules, including neoepitopes. Therefore, abnormal gene expression or immunogenic mutations can induce a T-cell-dependent immune response [41, 62]. Theoretically, neoantigens can potentially emerge during reprogramming, long-term cultivation, or differentiation of iPSCs into the desired cell type. Here we report that autologous as well as allogeneic T-cells responded to the same extent to isogeneic fibroblasts and iPS-fibro. Thus, the iPS-fibro of both donors did not accumulate any protein alterations that could be recognized by pre-existing reactive T-cells. We also did not find increased expression of *HORMAD1* and *ZG16* genes in iPS-fibro. These genes were previously correlated with immunogenicity properties of syngeneic and autologous iPSC-derivatives in mice [60, 61].

Although NK-cell activity against undifferentiated iPSCs was previously examined in a number of studies [63–66], the NK-cell response to differentiated iPSC-derivatives is poorly investigated. Few studies have reported NK-cell-mediated killing of murine iPSC-derived cells both in vitro [67] and in vivo models [68]. Human iPSC-derivatives were studied mainly upon engineering the immune-evasive HLA-I-deficient hPSCs. A priori, these studies were performed in the allogeneic mode. Suzuki et al. detected the same NK-cell degranulation against allogeneic HLA-depleted and wild-type iPSC-derived platelets [19]. The vascular smooth muscle cells (VSMCs) derived from HLA-ABC^−/−^ ESCs triggered degranulation and NK-cell-mediated cytotoxicity to the same extent as wild-type VSMCs [23]. Petrus-Reurer et al. reported the same NK-cell responses to HLA-I positive and HLA-I negative ESC-derived RPEs [30]. We also observed that the NK-cell response did not differ between HLA-I positive iPS-fibro and HLA-I negative ΔiPS-fibro. Moreover, we obtained the same result for allogeneic and autological modes. Thus, iPSC-derivatives were vulnerable to NK-cells regardless the HLA-I status.

The HLA-I molecules on the cell surface defend the cell from an NK-cell’s attack, but they are not alone in regulating immune tolerance. The cell also has other NK-cell receptor ligands contributing differentially to immune tolerance. The proper balance between inhibitory and activating ligands makes the target cell invisible to NK-cells [69]. The anergy of NK-cells is possible only with a weak positive signal coming from activating receptors and an active negative signal coming from inhibitory receptors. Therefore, we proposed that the imbalance of inhibitory and activating ligands in iPS-derived cells may be a reason for NK-cell activation.

In the above-mentioned studies, the authors didn’t focus on NK-cell response to wild-type hPSC-derivatives. Rather, they described the absence of hypersensitivity to NK-cells toward HLA-negative cells. At least two groups reported decreased level of NK-cell activating ligands such as CD155 and MICA/MICB [19, 30]. They assumed that the lack or deficiency of an NK-cell activation signal on HLA-I depleted hPSC-derivatives might make them resistant to NK-cell-mediated cytotoxicity. As opposed to other authors, we used parental fibroblasts as a negative control for NK-cell reaction. This comparison enabled us to detect the severe susceptibility of iPSC-derivatives to NK-cell effector functions.

Since autologous fibroblasts induced NK-cell anergy, we accepted them as the “gold standard” with the proper balance of NK-cell ligands. Transcriptome analysis revealed that major inhibitory ligands’ genes were underexpressed while some activating ligands’ genes were overexpressed in iPS-fibro compared to parental fibroblasts. Hence, the imbalance was determined simultaneously by two factors: low intensity of inhibitory signals and elevated intensity of activating signals.

Notably, we did not detect statistical differences in the expression of ligands for activating NK-cell receptors between wild-type iPS-fibro and HLA-I deficient ΔiPS-fibro. These results indicate that the inactivation of the *B2M* gene did not affect the pathways related to the regulation of NK-cell responses. Moreover, a trend toward the predominance of activating ligands was also observed in independently obtained samples. The expression level of ligands for DNAM-1 (*NECTIN2*, *PVR*), CRTAM (*CADM1*), and CD27 (*CD70*) receptors was elevated in all analyzed publicly available fibroblast-like cells. Similarly, some genes of adhesion molecules (*ICAM-1* and *VCAM-1*) responsible for establishing tight cell-to-cell contacts were also overexpressed in third-party iPSC-derivatives. In one case, there was also a significant decrease in the expression of inhibitory ligand *HLA-A* and *B2M* gene necessary for the formation of all HLA class I molecules. Due to the high level of correlation between our iPS-fibro, including ΔiPS-fibro, and independent iPSC-derived fibroblast-like cells, we suppose that the imperfect microenvironment during in vitro differentiation affected proper balance of ligands for NK-cell receptors. Since each cell type expresses its own set of proteins, for clinical practice it would be necessary to determine the expression patterns of ligands for NK-cell receptors.

A possible reason for the disturbed balance of NK-cell ligands in iPS-fibro may be insufficient cell maturity. We showed that at early passages, iPS-fibro retained a relatively low expression of HLA class I molecules typical for undifferentiated cells. In the process of cultivation and passaging, the expression of HLA-I molecules in iPS-fibro almost reached the level common for parental somatic cells. This theory is consistent with data received on ESC-derived RPEs [30]. The authors noted that HLA-I were induced by differentiation. Nevertheless, even though prolonged passaging compensated for the shortage of inhibitory signals in iPS-fibro, we demonstrated that it was insufficient to suppress the NK-cell response due to the excessive expression of major activating ligands.

Since iPS-fibro, regardless of their maturity, conducted excessive signals from activating NK-cell ligands, we studied whether it is possible to amplify signals from inhibitory NK-cell ligands. IFNγ is an important pro-inflammatory cytokine, which is produced mainly by activated T-cells and NK-cells, and impacts on immune and non-immune cells, including during transplantation [70]. A unique feature of this cytokine is the ability to enhance the expression of HLA molecules [55]. Due to the important biological role of IFNγ, we used it as an external signal to change the expression pattern of inhibitory and activating NK-cell ligands in iPS-fibro. We demonstrated that IFNγ treatment significantly increased the expression of inhibitory molecules, such as major inhibitory NK-cell ligand HLA-I and minor ones, including *CEACAM1*, *LGALS9*, *CD274* and *IDO*. Meanwhile, gene expression of activating ligands was slightly affected. Thus, IFNγ treatment tipped the balance, and HLA-I molecules became capable of inhibiting the NK-cell degranulation and NK-cell-mediated cytotoxicity against autologous and allogeneic iPS-fibro. Recently, it was shown that IFNγ could enhance the immunosuppressive properties of mesenchymal stem cells in a model of experimental renal fibrosis [71]. Our results indicate that IFNγ may also be a promising candidate for protecting autologous cell products from an immune response mediated by NK-cells.

Generally, the role of NK-cells in solid organ transplantation remains quite controversial [72]. There is evidence that some NK-cell subsets may play a role in the regulation of allograft tolerance, and NK-cells are nevertheless involved in T-cell-mediated and antibody-mediated allograft rejection [73]. In the absence of immunosuppressive therapy, which affects cytotoxic activity and adjusts degranulation properties, activated NK-cells produce IFNγ that may contribute to the development of chronic inflammation and the attraction of T-cell-mediated responses [74]. These immune processes may further complicate the use of autologous iPSC-derivatives in clinical practice. Currently three in-human transplants of autologous iPSC-derived cells have taken place [59, 75, 76]. Though these patients did not receive immunosuppression, it was reported that no one suffered from side effects. Still, even a possible chance of immune rejection raises concerns regarding the transplantation of autologous iPSC-derivatives without immunosuppressants. In this regard, different types of iPSC-derivatives must be tested for the proper balance of ligands for NK-cell receptors in order to avoid undesirable immune cell responses.

## Conclusions

The first successful transplantations of autologous iPSC-derivatives contribute to the development of personalized regenerative medicine [58]. However, the issue of complete immune tolerance of autologous iPSCs is not yet fully resolved. In contrast to earlier reports [39, 40], our study shows that cells differentiated from autologous iPSCs can be recognized as “non-self” by NK-cells rather than by T-cells. While the parental fibroblasts used for reprogramming did not disrupt the anergy of autologous NK-cells, fibroblast-like cells derived from iPSCs triggered the cytotoxic activity of NK-cells regardless of their HLA-I status. These results once again prove that the regulation of NK-cell activation is more complex than the “missing-self” hypothesis and depends on the interaction of inhibitory and activating NK-cell receptors. We showed that iPSC-derivatives had a disturbed balance of NK-cell ligands. Compared to the parental fibroblasts, the balance in iPS-fibro was shifted toward activating signals due to reduced expression of inhibitory molecules and increased expression of activating molecules. Maturation of iPS-fibro compensated for their deficiency in inhibitory NK-cell ligands. However, long-term culture did not affect the activating molecules, so it could not attenuate NK-cell response to more mature iPSC-derived cells. Pretreatment of iPS-fibro with the proinflammatory cytokine IFNγ boosted the expression of inhibitory molecules, thereby balancing ligands for NK-cell receptors. Our results suggest that even autologous cells differentiated from iPSCs need to be confirmed immunotolerant for future transplants.

## Supplementary Information

**Additional file 1.** Supplemental Experimental Procedures.

**Additional file 2.** Supplemental Figures.

**Additional file 3. **Transcriptomic data on selected genes encoding ligands for NK-cell receptors and other molecules necessary for NK-cell activation (GO:0030101).

## Data Availability

Publicly available datasets analyzed in the present study can be downloaded from NCBI GEO data repository under accession numbers GSE61390, GSE62772, GSE73211. Raw and processed datasets obtained in the present study can be downloaded from NCBI GEO under accession GSE212796. Supporting data are available from the corresponding author upon reasonable request.

## Abbreviations

B2M: Beta-2-microglobulin
CM: Cardiomyocytes
ESCs: Embryonic stem cells
HLA: Human leukocyte antigens
hPSCs: Human pluripotent stem cells
IFNγ: Interferon gamma
iPSCs: Induced pluripotent stem cells
LDH: Lactate dehydrogenase
NK: Natural killer
RPE: Retinal pigment epithelium
