# Supplementary material for: iPSC-derived cells lack immune tolerance to autologous NK-cells due to imbalance in ligands for activating and inhibitory NK-cell receptors
Source: Stem Cell Res Ther. 2023 Apr 11;14:77. doi: 10.1186/s13287-023-03308-5 (PMC10088155; doi:10.1186/s13287-023-03308-5)
Supplement: Supplementary file 1 — Additional file 1. Supplemental Experimental Procedures. [file 13287_2023_3308_MOESM1_ESM.docx]

# Additional file 1: Supplemental Experimental Procedures.

**Fibroblast isolation from human skin biopsies.**

After signing the informed consent, patients underwent skin biopsy of the forearm. The biopsy was placed in a drop of medium on a Petri dish and cut into small pieces (about 1 mm in size) using a sharp sterile scalpel. The obtained pieces were placed in separate 35 mm Petri dishes in 3 ml of culture medium and pressed with a sterile coverslip (Menzel Glasser). The medium was changed twice a week. After 3 weeks, fibroblasts were detached and passaged using 0,25 % EDTA solution (Gibco).

**Generation of iPSCs.**

2x10^5^ human skin fibroblasts were plated to 35 mm culture dish. Non-integrative reprogramming of human skin fibroblasts was performed with CytoTune™-iPS 2.0 Sendai Reprogramming Kit (ThermoFisher Scientific) according to manufacturer's instructions. On day 21 after transfection, the iPSCs clones were manually picked and cultivated as individual cell lines.

**Analysis of pluripotency markers.**

To determine the expression of pluripotent markers, iPSCs were immunostained for NANOG, TRA-1-81, and SSEA-4 as described previously [1]. Additionally, RT-PCR was performed to confirm OCT4, SOX2, SALL4, and DPPA5 gene expression in iPSCs.

**Embryoid bodies (EBs) formation.**

EBs were formed using Aggrewell^TM^400 24-well plates (STEMCELL Technologies Inc). The next day EBs were transferred to Ultra-Low Attachment Plates (Corning) and cultured in EB medium containing DMEM/F12 medium (PanEco) supplemented with 15% KO SR (Invitrogen), 5% FBS (Gibco), 0.1 mM β-mercaptoethanol (Sigma-Aldrich), and 1% NEAA (PanEco). The culture medium was replaced every 3 days. On day 12, EBs were collected and plated on a gelatin-coated plate and cultured for the next 10 days using the same medium. On day 23, EBs were fixed with 4% PFA for 20 min at RT-and immunostained for CK18, CD31 and HNF4A, with specific markers of ectoderm, mesoderm, and endoderm, respectively.

**Karyotyping.**

The preparation of metaphase chromosomes and G-banding was performed according to standard protocol [2]. At least 20 metaphase spreads were analysed at a minimal resolution of 400 bands.

**iPS-RPE differentiation.**

iPSCs were differentiated into iPS-RPE as described previously [3]. Terminally differentiated cells were stained for Tight junction protein 1 (ZO-1) and Bestrophin-1 (Best1).

**iPS-CM differentiation.**

iPSCs were differentiated into iPS-CM using STEMdiff™ Cardiomyocyte Differentiation Kit (Stem Cell Technologies). The effectiveness of differentiation was assessed by morphological criteria and the presence of clusters of contracting cells. Terminally differentiated cells were stained with an antibody to the marker of cardiomyocytes – cardiac troponin T (cTnT).

**Selection of the key fibroblasts and iPSC markers.**

**scRNA-seq reads of human skin fibroblasts and reprogrammed iPSC were downloaded from the ArrayExpress Repository (dataset accession number is E-MTAB-10060) [4]. Raw gene-barcode matrices were obtained by aligning reads to the 10X Genomics GRCh38 reference genome (refdata-Gex-GRCh38-2020-A) using CellRanger software (v. 4.0.0) [5]. R packages Seurat (v. 4.2.0) and scran (v. 1.22.1) [6,7] were used for cell filtration, normalization, principal component analysis, variable genes search, clustering analysis, and Uniform Manifold Approximation and Projection (UMAP) dimensional reduction. Filtering was conducted by removing cells with a small number of unique molecular identifiers (UMIs) and detected genes, and with a high percentage of mitochondrial genes. Skin fibroblasts markers were selected based on the following criteria: each marker gene must be expressed by at least 40% fibroblast cells and by less than 1% iPSCs. Similarly, iPSC markers were selected. In total, 51 marker genes were identified (Additional File 2: Fig.S6).**

**References.**

1. Shuvalova LD, Davidenko AV, Eremeev AV, et al. Generation of induced pluripotent stem cell line RCPCMi008-A derived from patient with spinocerebellar ataxia 17. *Stem Cell Res*. 2021; 54:102431.
2. Shutova MV, Chestkov IV, Bogomazova AN, Lagarkova MA, Kiselev SL. Generation of iPS Cells from Human Umbilical Vein Endothelial Cells by Lentiviral Transduction and Their Differentiation to Neuronal Lineage. In: Ye K, Jin S, editors. Human Embryonic and Induced Pluripotent Stem Cells. *Springer Protocols Handbooks: Humana Press*; 2011. p. 133–149
3. Brandl C. Generation of Functional Retinal Pigment Epithelium from Human Induced Pluripotent Stem Cells. In: Weber BHF, Langmann T, editors. Retinal Degeneration. *Methods in Molecular Biology, vol 1834. New York: Humana*; 2019. p. 87–94.
4. Parkinson H, Kapushesky M, Shojatalab M, et al. ArrayExpress--a public database of microarray experiments and gene expression profiles. *Nucleic Acids Res*. 2007;35(Database issue):D747-D750.
5. Zheng GX, Terry JM, Belgrader P, et al. Massively parallel digital transcriptional profiling of single cells. *Nat Commun*. 2017;8:14049.
6. Stuart T, Butler A, Hoffman P, et al. Comprehensive Integration of Single-Cell Data. *Cell*. 2019;177(7):1888-1902.e21.
7. Lun AT, McCarthy DJ, Marioni JC. A step-by-step workflow for low-level analysis of single-cell RNA-seq data with Bioconductor. *F1000Res*. 2016;5:2122.

**Supplementary Table S1. List of antibodies used in this study.**

| **Antibody** | **Catalogue number** | **Dilution** | **Manufacturer** |
| --- | --- | --- | --- |
| FITC anti-human  beta-2-microglobulin | #2181520 | 1:500 | Sony Biotechnology |
| APC anti-human HLA-ABC | #2157045 | 1:200 | Sony Biotechnology |
| FITC anti-human CD73  (Ecto-55'-nucleotidase) | #2320075 | 1:200 | Sony Biotechnology |
| Alexa Fluor® 647  anti-human CD90 (Thy1) | #2240575 | 1:200 | Sony Biotechnology |
| APC anti-human CD105 | #2216040 | 1:200 | Sony Biotechnology |
| Brilliant Violet 785™  anti-human CD8 | #2323700 | 1:1000 | Sony Biotechnology |
| PerCP/Cy5.5 anti-human CD3 | #2186680 | 1:333 | Sony Biotechnology |
| PE anti-human CD69 | #2154530 | 1:250 | Sony Biotechnology |
| APC anti-human CD56 (NCAM) | #2191550 | 1:250 | Sony Biotechnology |
| PE/Dazzle™ 594 anti-human CD107a (LAMP-1) | #2243230 | 1:1000 | Sony Biotechnology |
| Ultra-LEAF™ Purified anti-human CD314 (NKG2D) | #320813 | 1:100 | Biolegend |
| FITC anti-human CD226 (DNAM-1) | #2285520 | 1:50 | Sony Biotechnology |
| Mouse IgG - Isotype Control | #Ab37355 | 1:500 | Abcam |
| Mouse anti-human  Nanog homeobox | PCRP-NANOGP1-2D8 | 1:40 | DSHB |
| Mouse anti-human TRA-1-81 | # 4745S | 1:40 | CellSignalling |
| Mouse anti-human SSEA-4 | #MC81370 | 1:40 | DSHB |
| Mouse anti-human cytokeratin 18 | #M7010 | 1:100 | Agilent DAKO |
| Rabbit anti-human CD31 | #Ab28364 | 1:100 | Abcam |
| Rabbit anti-human HNF4A | # Ab92378 | 1:100 | Abcam |
| Mouse anti-ZO-1 | #33-910 | 1:100 | Invitrogen |
| Rabbit anti-Bestrophin/BEST1 | #Ab14927 | 1:100 | Abcam |
| Mouse anti-troponin T cardiac | #4T19 | 1:1000 | Hytest |
| Goat anti-mouse igG (H+L), Alexa Fluor 555 | #A21422 | 1:800 | ThermoFisher Scientific |
| Goat anti-rabbit igG (H+L), AlexaFluor 488 | #A11008 | 1:800 | ThermoFisher Scientific |

**Supplementary Table S2. List of primers used in this study.**

| **Target** | **Forward primer 5’-3’** | **Reverse primer 5’-3’** |
| --- | --- | --- |
| B2M (seq) | CTGGCTTGGAGACAGGTGACGGTC | CGAGATCCAGCCCTGGACTAGC |
| M13 (seq) | GTTGTAAAACGACGGCCAGTG | AGCGGATAACAATTTCACACAGGA |
| OCT4 | CCTTCGCAAGCCCTCATTTC | AACCACACTCGGACCACATC |
| SOX2 | AACCAGCGCATGGACAGTTA | GACTTGACCACCGAACCCAT |
| SALL4 | TGGCGGAGAGGGCAAATAAC | ATGCTGAAGAACTCCGCACA |
| DPPA5 | AAGATGGGAACTCTCCCGGC | GCAAGTTTGAGCATCCCTCGC |
| GAPDH (Tm 60) | GAAGGTGAAGGTCGGAGTCA | GTTGAGGTCAATGAAGGGGTC |
| GAPDH (Tm 66) | TCAAGAAGGTGGTGAAGCAGGCGT | AAGGTGGAGGAGTGGGTGTCGCTG |
| B2M (qPCR) | AGATGAGTATGCCTGCCGTGTG | GCGGCATCTTCAAACCTCCA |
| HLA-A | TGTTCTAAAGTCCGCACGCA | CGGGACACGGATGTGAAGAA |
| HLA-B | CTAGCAGTTGTGGTCATCGGA | TGCATCTCAGTCCCTCACAAG |
| HLA-C | ACCCACCCGGACTCACATTCTC | ATACCTCATGGAGTGGGAGCAGGC |
| MICA | ACTCACCCTGTGCCCTCTGGGAAA | GCAGCAGCAACAGCAGAAACATGGA |
| ULBP1 | CAGAGAAGTGGGAGAAGAACAGG | GCCAGAGAGGGTGGTTTTGTT |
| ULBP3 | GGGAGAAGGATAGCGGACTGACC | GGTGGGTGGTGCTGTGGGTTC |
| CD112 | GGTGGAGGACGAGGGCAACTAC | GCTTGGTTCTTGGGCTTGGCTATG |
| CD155 | CCTCAGCTAATGGGCATGTCTCCT | CAGTCCCGACGCTGTCACCTTG |
