# Supplementary material for: iPSC-derived cells lack immune tolerance to autologous NK-cells due to imbalance in ligands for activating and inhibitory NK-cell receptors
Source: Stem Cell Res Ther. 2023 Apr 11;14:77. doi: 10.1186/s13287-023-03308-5 (PMC10088155; doi:10.1186/s13287-023-03308-5)
Supplement: Supplementary file 2 — Additional file 2. Supplemental Figures. [file 13287_2023_3308_MOESM2_ESM.docx]

# Additional file 2: Supplemental Figures.


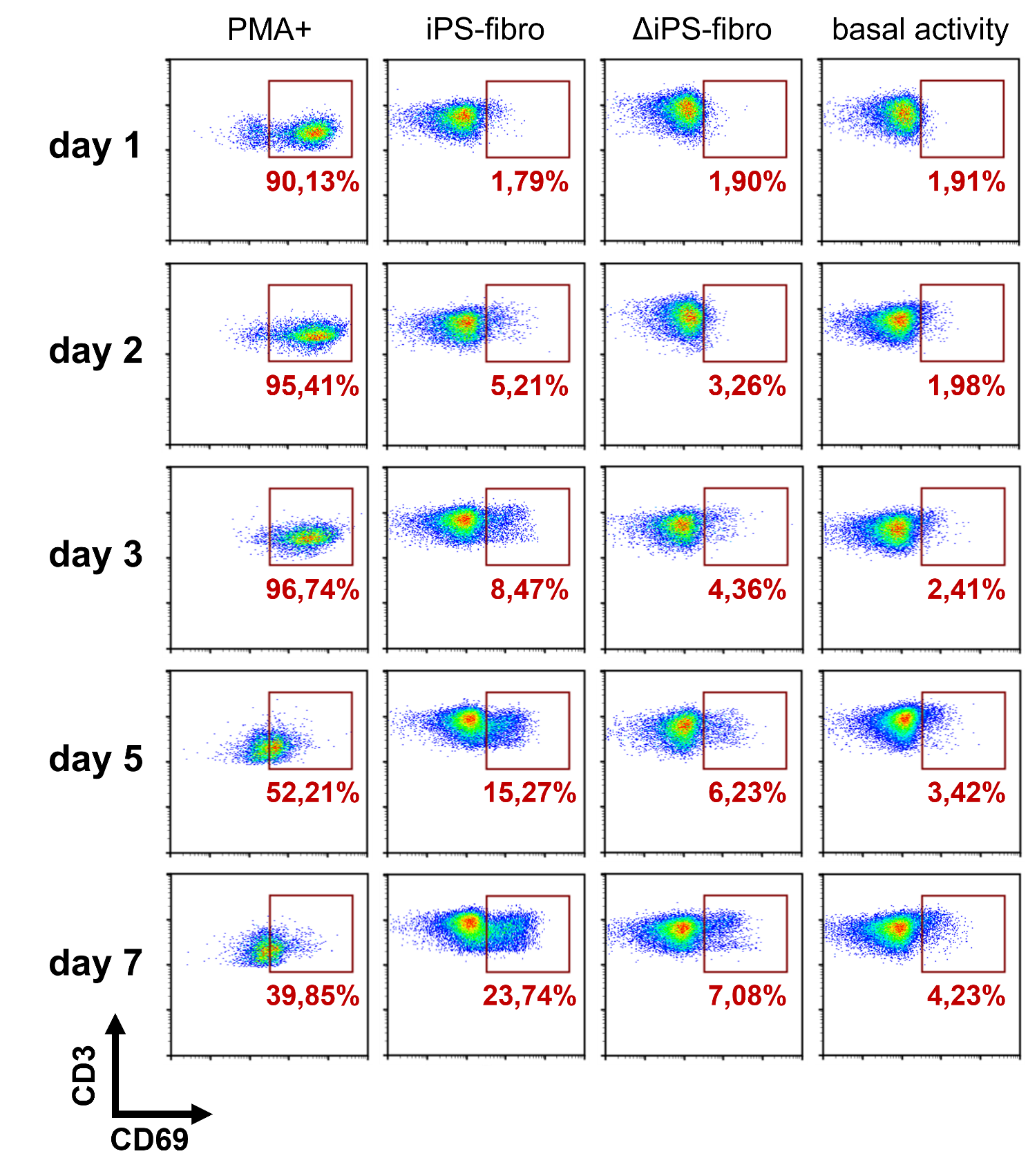


**Supplementary Figure S1.** **Time-course of T-cell activation against iPS-fibro and ΔiPS-fibro.** T-cells treated with phorbol myristate acetate (PMA) were used as a positive control. Unstimulated T-cells were used as a negative control.


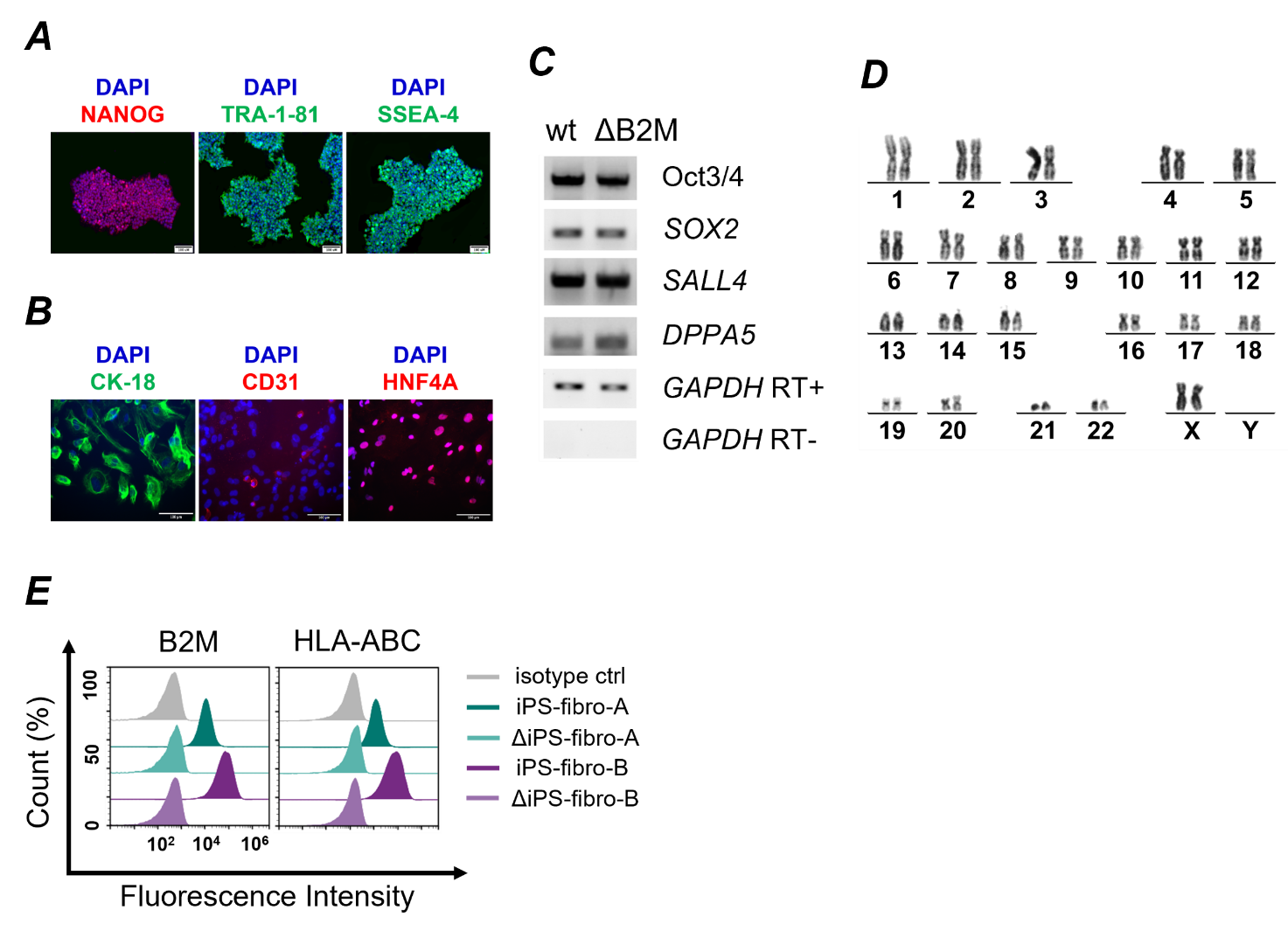


**Supplementary Figure S2. Characterization of cell line ΔiPSC-B.** (A) Immunohistochemical staining for pluripotency markers (NANOG, TRA-1-81, SSEA/4) in ΔiPSC-B (Scale bars, 100 μm). (B) Layer markers, namely ectoderm (CK18), mesoderm (CD31), and endoderm (HNF4A), were expressed by ΔiPSC-B derivatives after spontaneous in vitro differentiation (Scale bars, 100 μm). (C) RT-PCR confirmed a high amount of OCT3/4, SOX2, SALL4, and DPPA5 mRNA expression in iPSC-B and ΔiPSC-B. Full-length gels are shown in Supplementary Figure S3. (D) G-banding analysis of ΔiPSC-B showed a normal karyotype. (E) Flow cytometry analysis of B2M and HLA-ABC expression in iPS-fibro and ΔiPS-fibro.


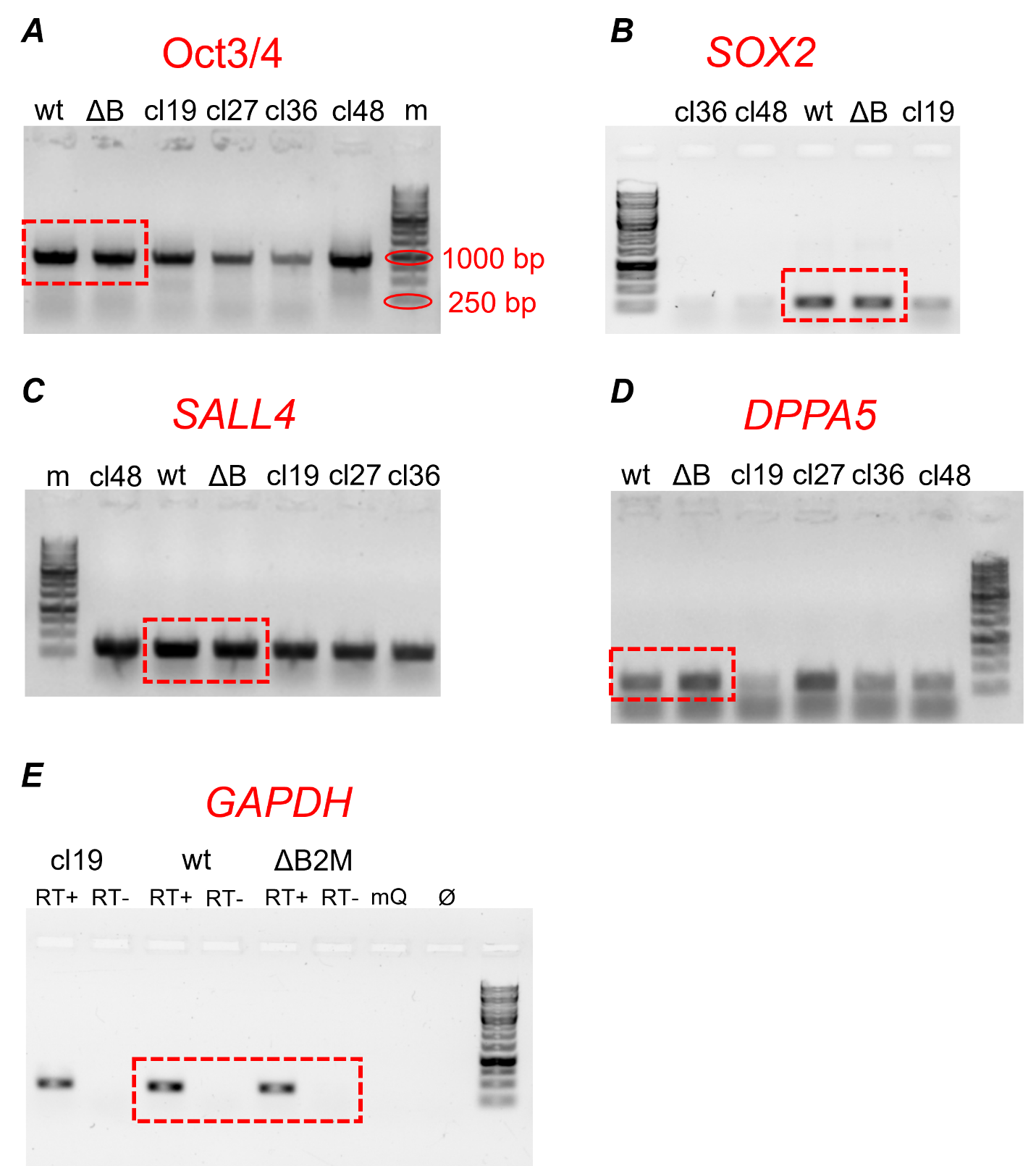


**Supplementary Figure S3.** **Full-length gels of RT-PCR analysis for detection of the pluripotency markers: Oct3/4 (A), SOX2 (B), SALL4 (C), DPPA-5 (D).** GAPDH RT+ (E) was used as a positive control of reverse transcription reaction. GAPDH RT- (E) was used as a control for genomic DNA contamination. The cropped area is marked with a red dotted line.


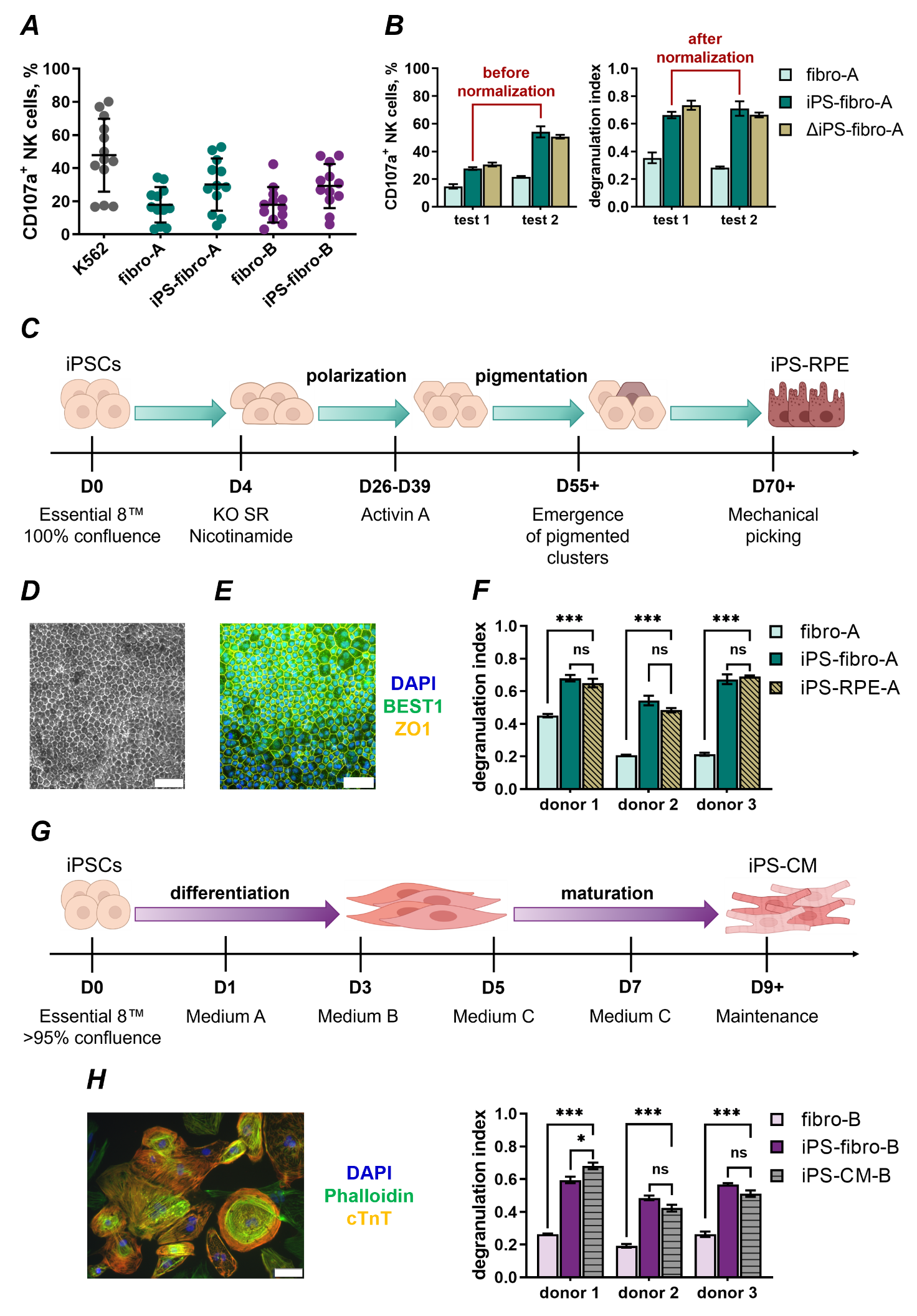


**Supplementary Figure S4. In vitro NK-cell response to various iPSC-derivatives.** (A) Dot plot (N=12) illustrating the high variation in the CD107a mobilization assay. The dots represent the mean ± SD. (B) Comparison of CD107a^+^ NK-cells and the degranulation index of one allogeneic donor before and after normalization by the level of NK-cell response to the K562 cell line. The bars represent the mean ± SEM. (C) Schematic diagram of the experimental protocol for iPS-RPE differentiation. (D) Morphology of iPS-RPE (Scale bars, 25 μm). (E) Immunohistochemical staining for RPE markers (BEST-1, ZO-1) in iPS-RPE (Scale bars, 25 μm). (F) Allogeneic NK-cells demonstrated significantly higher degranulation against iPS-RPE compared to dermal fibroblasts. Each bar represents the mean ± SEM; ***P < 0.001; two-way ANOVA. (G) Schematic diagram of the experimental protocol for iPS-CM differentiation. (H) Immunohistochemical staining for CM marker (cTnT) in iPS-CM (Scale bars, 25 μm). (I) Allogeneic NK-cells demonstrated significantly higher degranulation against iPS-CM compared to dermal fibroblasts. Each bar represents the mean ± SEM; *P < 0.05; ***P < 0.001; two-way ANOVA.


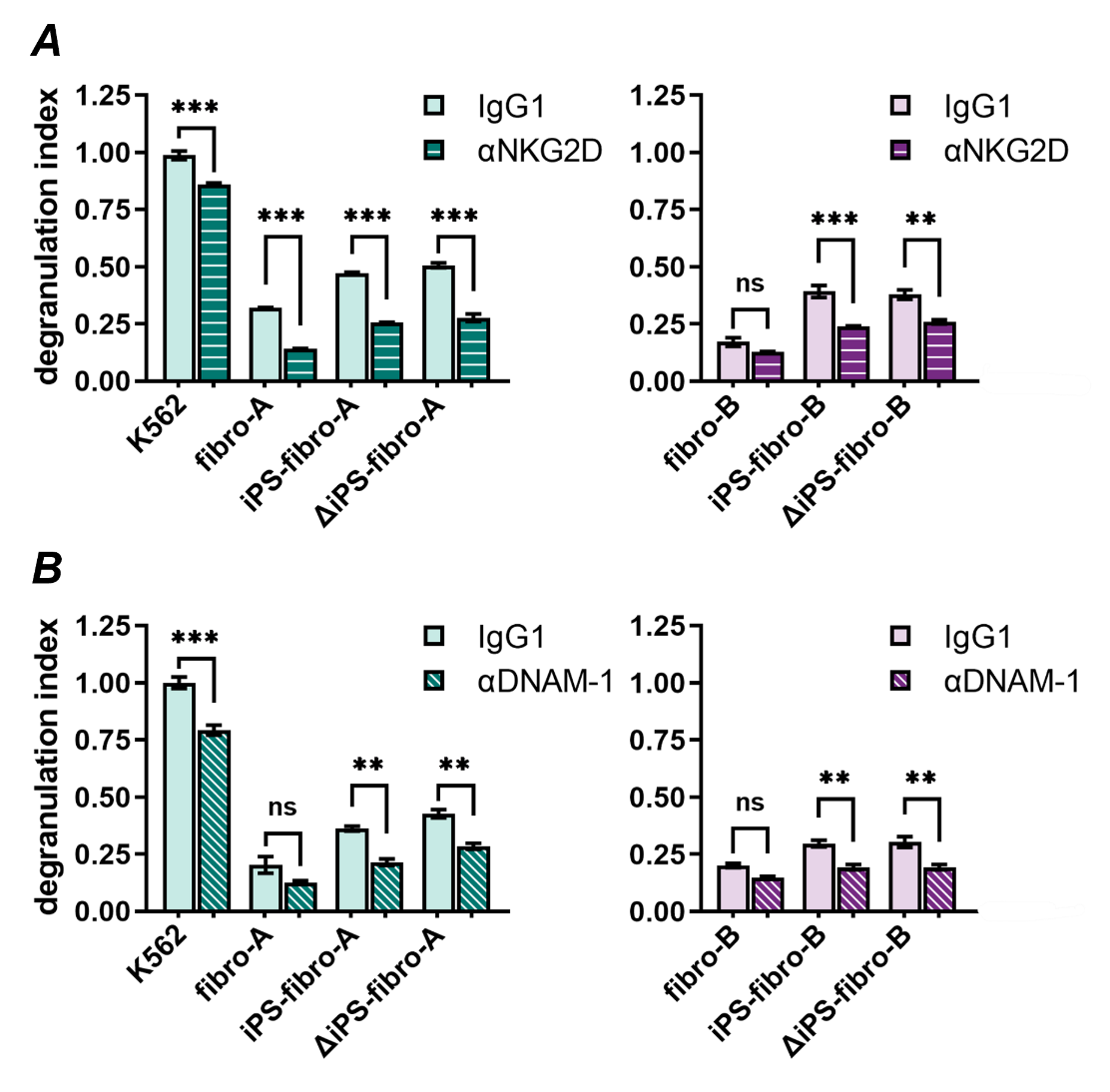


**Supplementary Figure S5. Blocking key activating NK-cell receptors diminished excessive degranulation against both wild-type and KO *B2M* iPSC-derived cells.** (A) Degranulation index of NK-cells incubated with isotype control (IgG1) or anti-NKG2D blocking antibodies (αNKG2D) at a concentration of 10 μg/ml. The bars represent the mean ± SEM; **P < 0.01; ***P < 0.001; ordinary one-way ANOVA. (B) Degranulation index of NK-cells incubated with isotype control (IgG1) or anti-DNAM-1 blocking antibodies (αDNAM-1) at a concentration of 10 μg/ml. The bars represent the mean ± SEM; **P < 0.01; ***P < 0.001; ordinary one-way ANOVA.


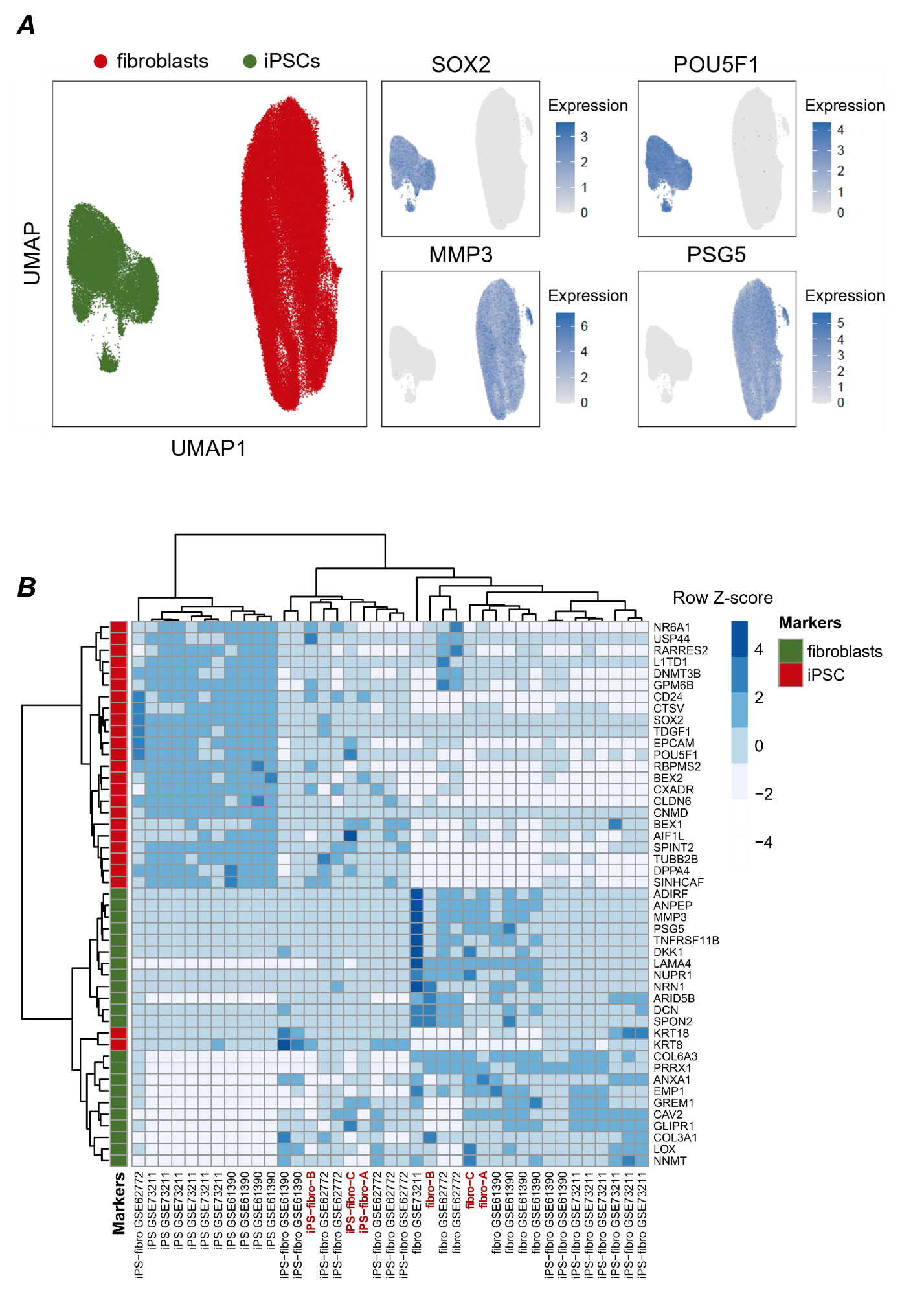


**Supplementary Figure S6. The transcriptomic signature of fibroblast-like iPSC-derivatives resembled dermal fibroblasts.** (A) Key markers typical for fibroblasts (indicated in green) and undifferentiated iPSCs (indicated in red) were identified using third-party scRNA-seq data. (B) Hierarchical clustering heatmap illustrating the expression of the key markers typical for fibroblasts (indicated in green) and iPSCs (indicated in red) in dermal fibroblasts, iPS-fibro and undifferentiated iPSCs. The expression values of each gene in a row are normalized by a row Z-Score.  Samples obtained in this study are indicated in red.


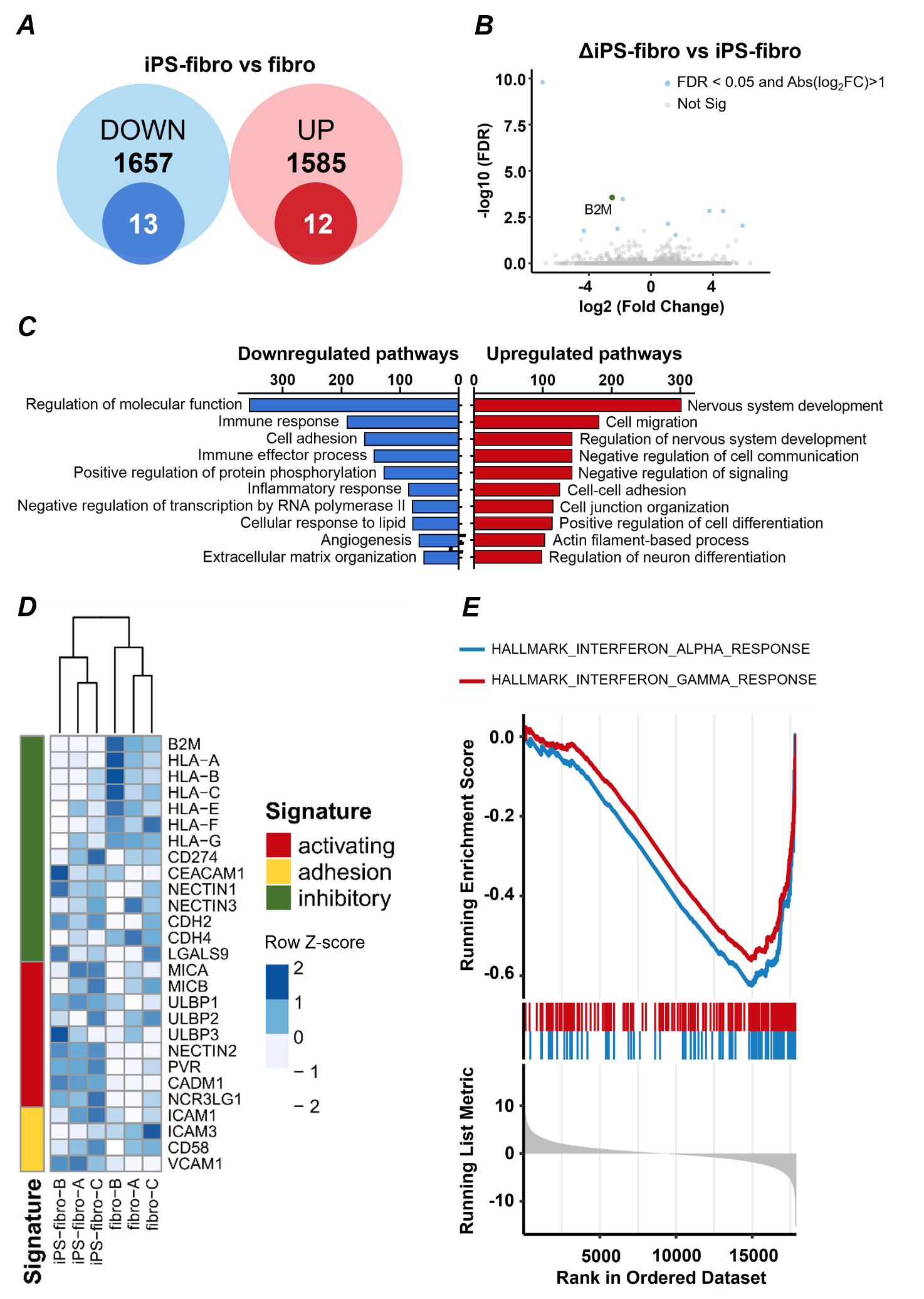


**Supplementary Figure S7. Differentially expressed genes in iPS-fibro.** (A) The number of differentially expressed genes in iPS-fibro with fold change >1.5 (FDR< 0.05). The inner circles illustrate differentially expressed genes encoding the ligands for NK-cell receptors or molecules necessary for NK-cell activation (GO:0030101). (B) Transcriptomic profiles of iPS-fibro and ΔiPS-fibro did not differ significantly, except for a few genes, in particular *B2M*. (C) Gene Ontology (GO) enrichment analysis of the differentially expressed genes in iPS-fibro. (D) Heatmap of the gene expression encoding the key ligands for NK-cell receptors and adhesion molecules. (E) GSEA analysis for interferon alpha and gamma response.


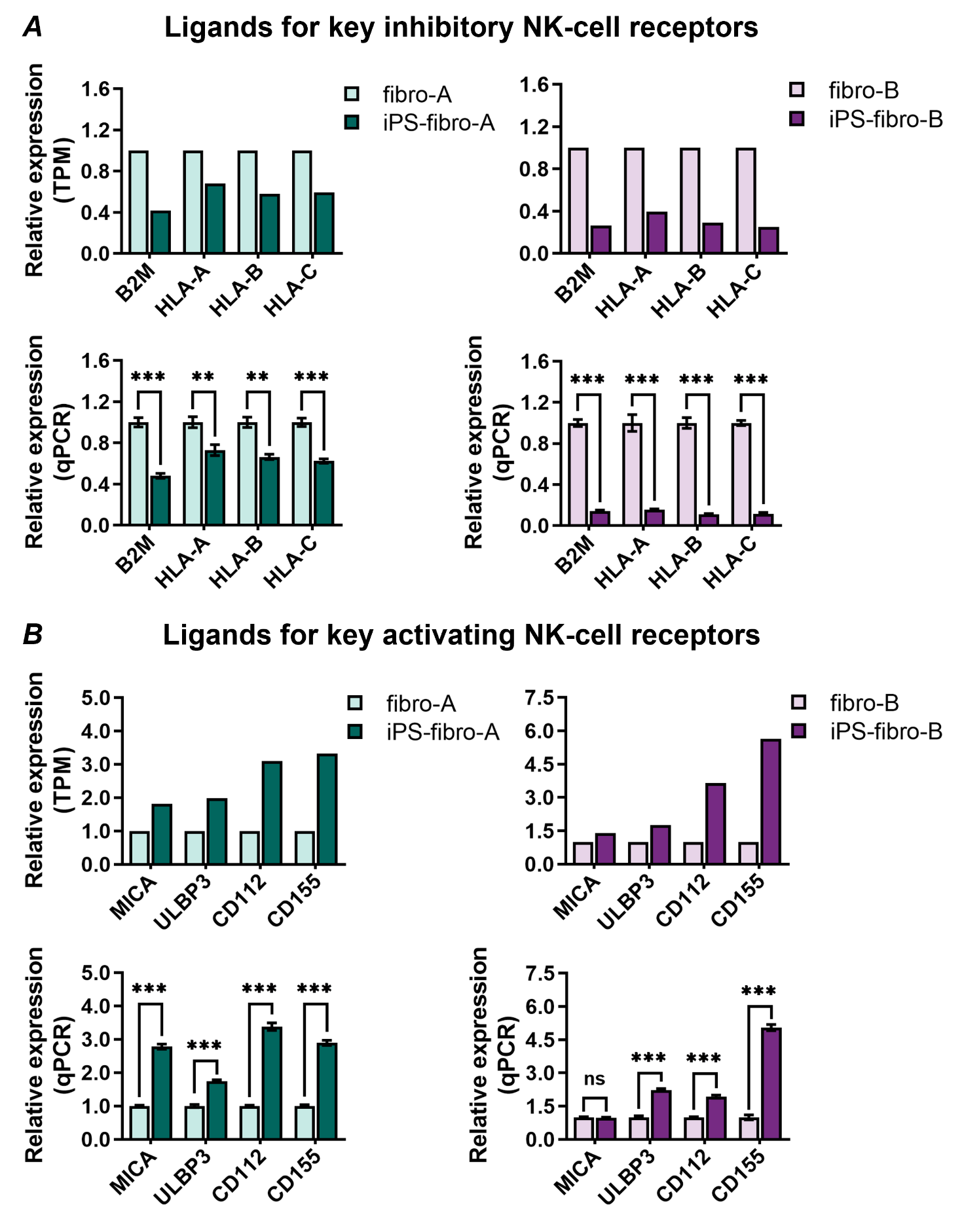


**Supplementary Figure S8. qPCR verification of selected ligands for NK-cell receptors.** (A) Comparison of RNA sequencing data (top) with qRT-PCR data (bottom) for genes encoding ligands for key inhibitory NK-cell receptors. The bars represent the mean ± SEM; **P<0.01; ***P < 0.001; two-way ANOVA. (B) Comparison of RNA sequencing data (top) with qRT-PCR data (bottom) for genes encoding ligands for key activating NK-cell receptors. Expression of all analyzed genes in iPS-fibro was normalized to isogeneic parental fibroblasts. The bars represent the mean ± SEM; ***P < 0.001; two-way ANOVA.

**
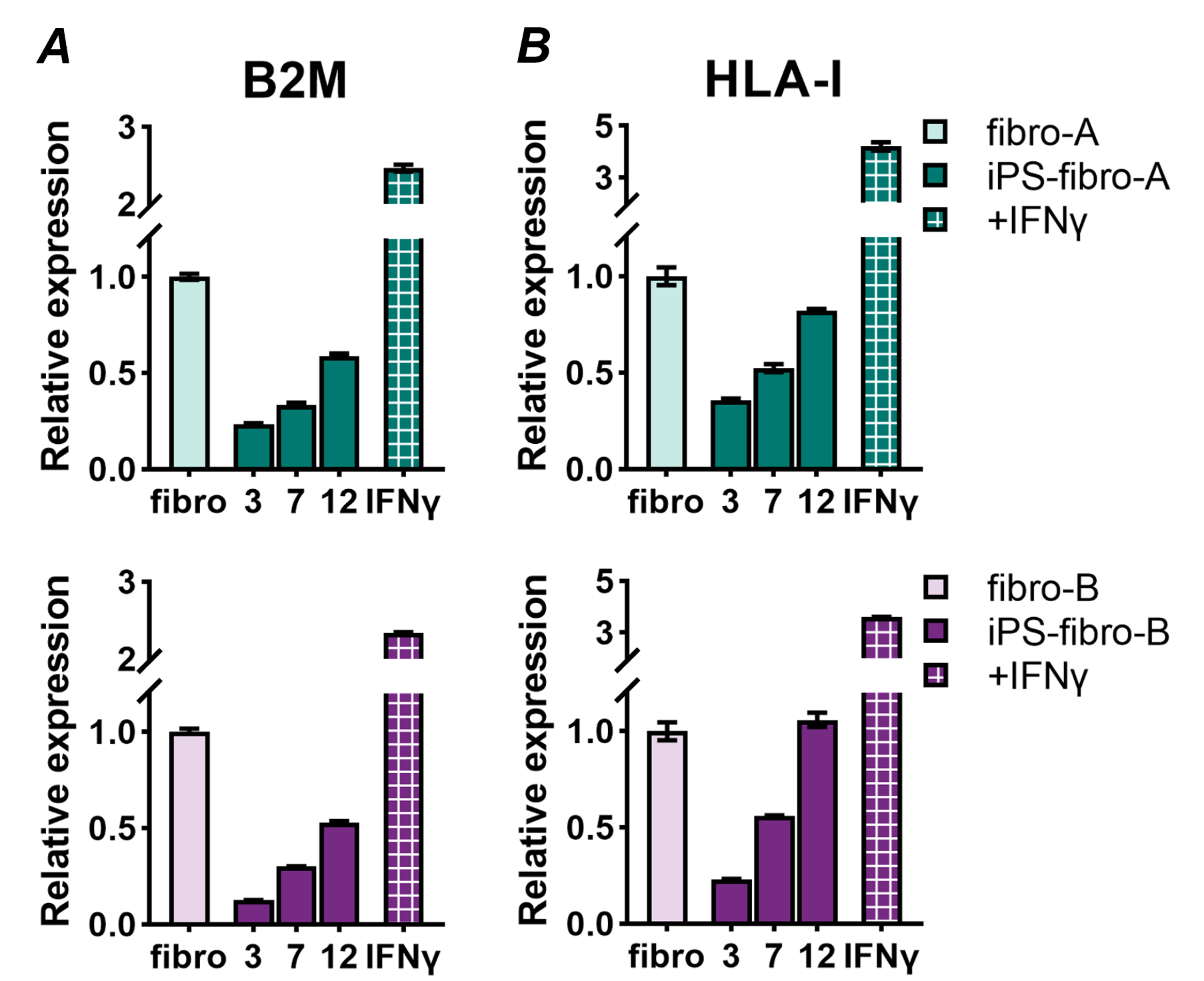
**

**Supplementary Figure S9. Comparison of B2M (A) and HLA-I (B) content during the passaging of iPS-fibro and after IFNγ stimulation.** The bars represent the mean ± SEM. The expression of analyzed proteins was measured by flow cytometry and normalized to isogeneic parental fibroblasts.


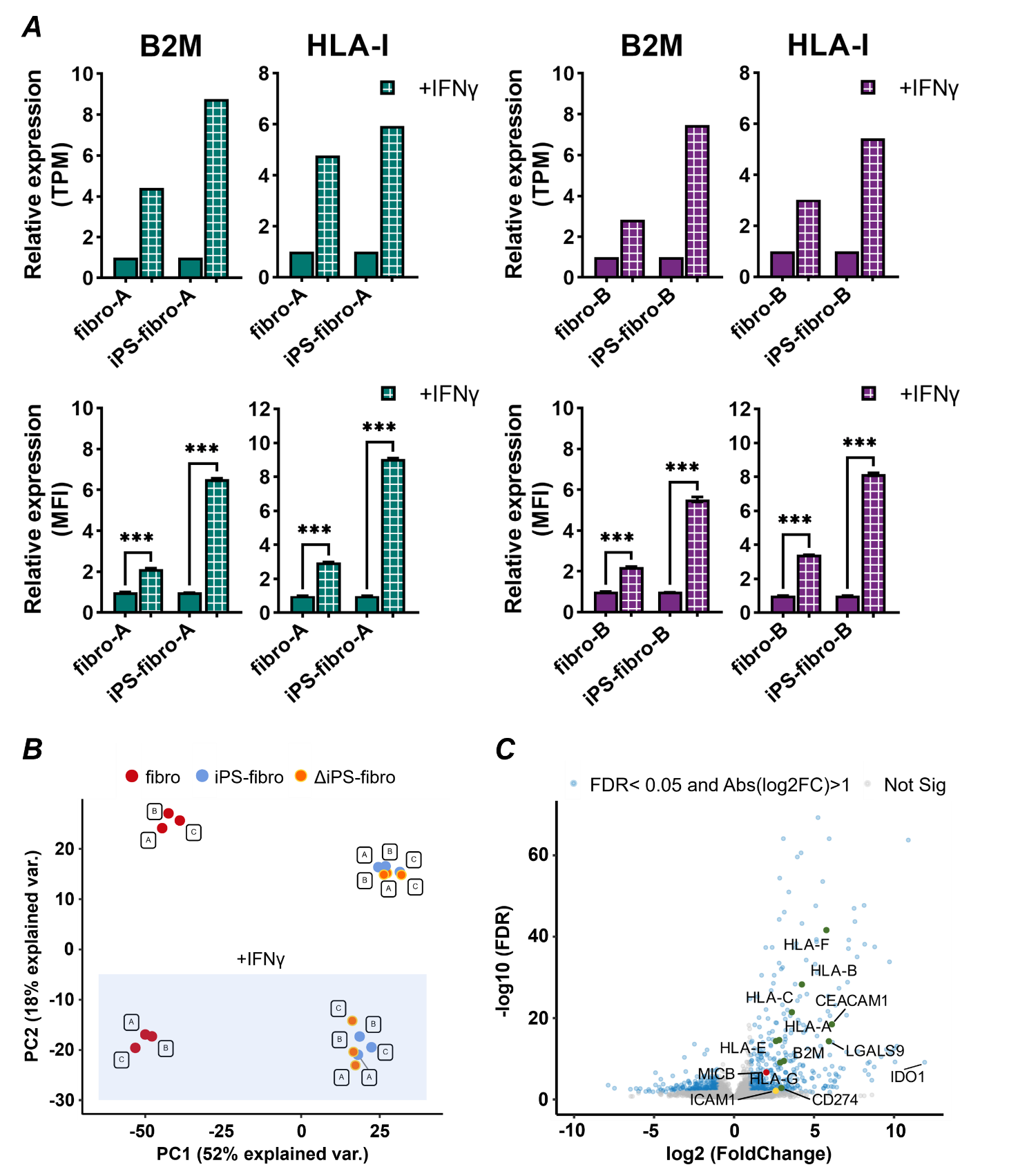


**Supplementary Figure S10. IFNγ stimulation brings the balance of NK-cell ligands to the proper state.** (A) Comparison of RNA sequencing data (top) with flow cytometry data (bottom) for B2M and HLA-I expression. Expression of all analyzed genes and proteins was normalized to intact controls. The bars represent the mean ± SEM; ***P < 0.001; two-way ANOVA. (B) PCA plots illustrating IFNγ exposure. (C) Volcano plot illustrating the differentially expressed genes in iPS-fibro after IFNγ stimulation. Genes encoding inhibitory NK-cell ligands indicated in green, genes encoding activating NK-cell ligands indicated in red, adhesion molecules indicated in yellow.
